# *In vitro* assembly and activity of an archaeal CRISPR-Cas type I-A Cascade interference complex

**DOI:** 10.1093/nar/gku120

**Published:** 2014-02-05

**Authors:** André Plagens, Vanessa Tripp, Michael Daume, Kundan Sharma, Andreas Klingl, Ajla Hrle, Elena Conti, Henning Urlaub, Lennart Randau

**Affiliations:** ^1^Prokaryotic Small RNA Biology Group, Max Planck Institute for Terrestrial Microbiology, D-35043 Marburg, Germany, ^2^Bioanalytical Mass Spectrometry Group, Max Planck Institute for Biophysical Chemistry, D-37077 Göttingen, Germany, ^3^Cell Biology and LOEWE Research Centre for Synthetic Microbiology, Philipps-Universität Marburg, D-35043 Marburg, Germany and ^4^Department of Structural Cell Biology, Max Planck Institute of Biochemistry, D-82152 Martinsried, Germany

## Abstract

Clustered Regularly Interspaced Short Palindromic Repeats (CRISPR)-CRISPR-associated (Cas) systems of type I use a Cas ribonucleoprotein complex for antiviral defense (Cascade) to mediate the targeting and degradation of foreign DNA. To address molecular features of the archaeal type I-A Cascade interference mechanism, we established the *in vitro* assembly of the *Thermoproteus tenax* Cascade from six recombinant Cas proteins, synthetic CRISPR RNAs (crRNAs) and target DNA fragments. RNA-Seq analyses revealed the processing pattern of crRNAs from seven *T. tenax* CRISPR arrays. Synthetic crRNA transcripts were matured by hammerhead ribozyme cleavage. The assembly of type I-A Cascade indicates that Cas3′ and Cas3′′ are an integral part of the complex, and the interference activity was shown to be dependent on the crRNA and the matching target DNA. The reconstituted Cascade was used to identify sequence motifs that are required for efficient DNA degradation and to investigate the role of the subunits Cas7 and Cas3′′ in the interplay with other Cascade subunits.

## INTRODUCTION

The coevolution of viruses with their prokaryotic hosts led to the development of specific and highly divergent antiviral prokaryotic immune systems. One complex group of adaptive immune systems that is widespread in bacterial and archaeal genomes is termed Clustered Regularly Interspaced Short Palindromic Repeats (CRISPR)-CRISPR-associated (Cas). Cells that harbor these systems can be immunized against the attack of viruses by the integration of a virus-derived genome fragment into the host genome (1). The genetic memory of previous infections is mediated by CRISPR loci, which consist of a series of short repeat sequences (typically 24–37 bp) that are separated by spacer sequences (2–4). Cas proteins are often encoded in proximity to the CRISPR loci and are key players during all phases of immunization and protection of the cell (5,6). In the first phase, the adaptation, the injected viral DNA is recognized and a fragment is inserted into the host CRISPR array (7–9). This activity is often dependent on a short conserved sequence (2–5 bp) defined as the protospacer adjacent motif (PAM) that flanks the original spacer sequence (termed protospacer) in the viral genome (10,11). The genetic imprint is activated by the transcription of the CRISPR into a long precursor-crRNA (pre-crRNA), which is typically processed by the endoribonuclease Cas6 into short crRNAs that are characterized by an 8-nt 5′-hydroxyl repeat tag, a complete spacer sequence and a 2′–3′ cyclic phosphate repeat end (12–18). During a repeated viral attack, the mature crRNAs can be incorporated into a large Cas ribonucleoprotein interference complex to target the viral DNA for degradation (19–21). 

These basic principles of CRISPR-Cas immunity are conserved, but careful computational and biochemical analyses of the differences among the executing interference machines, the composition of conserved Cas marker proteins and the nature of the targeted nucleic acids led to the identification of three distinct major types and several subtypes of CRISPR-Cas systems (5,22). The type I CRISPR-Cas systems can be further divided into six different subtypes (subtypes I-A to I-F), and the respective interference complex is termed Cascade (19). In type III systems, interference is executed by the Csm (subtype III-A, targeting DNA) or Cmr complex (subtype III-B, targeting RNA) (23–25). In contrast, bacterial type II systems are characterized by the single large multifunctional protein Cas9, which is involved in both the maturation of crRNAs and the interference of DNA (26–28). 

First details of the Cascade structure and the molecular mechanism were obtained for type I-E systems of *Escherichia coli*. The I-E Cascade shows a seahorse-shaped architecture with a size of 405 kDa and is composed of the conserved subunits Cas6e, Cas7, Cas5e and the subtype-specific nucleic acid-binding proteins Cse1 and Cse2 (19,29,30). The helical backbone of I-E Cascade is formed of six Cas7 copies that are tightly bound to a mature crRNA (31,32). The I-E Cascade facilitates the base pairing of the bound crRNA with the complementary DNA by screening for the short PAM sequence, resulting in strand invasion of the RNA and additional displacement of the non-complementary strand to form the R-loop structure, which recruits Cas3 to degrade the targeted viral DNA (18,33–36). The effector protein Cas3 contains a DExH-like helicase domain (Cas3′) and a HD phosphohydrolase domain (Cas3′′), which are responsible for the unwinding of double-stranded DNA (dsDNA) and cleavage of single-stranded DNA (ssDNA) in dependence of adenosine triphosphate (ATP) and divalent metal ions, respectively (37–39). 

The type I CRISPR-Cas subtypes differ in Cas protein content, which implies divergent Cascade assembly strategies and functional differences. Comparative studies of these different Cascade complexes will help to gain insight into the evolution and propagation of CRISPR-Cas systems, the integration of one or multiple immunity systems into the cellular protein network and the adaptation mechanisms to diverse prokaryotic environments (5,40). Analysis of the CRISPR-Cas system of the crenarchaeon *Thermoproteus tenax* identified a type I-A Cascade module (*csa5*, *cas7*, *cas5a*, *cas3*′, *cas3*′′, *cas8a2*), essential genes for the adaptation of foreign DNA (*cas1*/*cas2*, *cas4*), a type III-A gene cluster, a second subset of a Cascade module and seven CRISPR loci spread throughout the genome (41,42). In *Sulfolobus solfataricus*, the type I-A Cascade sub-complex of Cas7 and Cas5a was identified and shown to bind crRNA and complementary ssDNA. The recombinant Cas7 proteins assembled into multimeric right-handed helical structures (43).

Here, we show the assembly of a complete type I-A Cascade from individual *in vitro-*produced Cas proteins, ribozyme-processed synthetic crRNAs and short protospacer DNA fragments. The strategy for the protein body assembly of the mature Cascade follows the co-refolding of insoluble recombinant I-A Cas proteins from bacterial inclusion bodies to recover the six protein complex. Synthetic crRNAs were created by *in vitro* transcription of crRNA constructs fused to *cis*-acting hammerhead ribozymes. The assembly of the Cascade ribonucleoprotein complex yielded active molecules that showed crRNA-specific DNA targeting and degradation. This *in vitro* assembly strategy allowed us to obtain insights into the Cascade assembly and DNA cleavage mechanism and to identify the PAM requirements for target degradation.

## MATERIALS AND METHODS

### Strains and growth conditions

Cells of *T. tenax* Kra1 (DSM 2078) grown heterotrophically in *Thermoproteus* medium (44) were a gift from R. Hensel (Essen). *E. coli* strains TOP10 (Invitrogen) and Rosetta2(DE3)pLysS (Stratagene) were cultured in LB medium at 37°C shaking at 200 rpm. For protein production, 1 mM isopropyl-β-d-1-thiogalactopyranoside (IPTG) was added to a growing culture (OD_600_: 0.6) and incubated for 4 h.

### Isolation of small RNAs, production of crRNAs and DNA substrates

For the preparation of *T. tenax* small RNAs (<200 nt), 0.1 g pelleted cells were lysed by homogenization and subsequently isolated according to the *mir*Vana™ miRNA Isolation Kit (Ambion). To generate synthetic crRNAs, forward and reverse complementary DNA oligonucleotides (cr5.2h or cr5.13h, respectively) were synthesized that contained a selected spacer sequence (spacer 5.2 or spacer 5.13) and were fused with the sequence of a minimal *cis*-acting hammerhead ribozyme at the 5′-end (Supplementary Table SI). The oligonucleotides were phosphorylated, hybridized and cloned under control of the T7 RNA polymerase promoter sequence (cr5.2h: BamHI/HindIII, cr5.13h: HindIII/EcoRI) into pUC19. The crRNA was prepared by run-off transcription in a reaction containing 40 mM HEPES/KOH, pH 8.0, 22 mM MgCl_2_, 5 mM dithiothreitol, 1 mM spermidine, 4 mM of ATP, CTP, GTP and UTP, 20 U RNase inhibitor, 1 µg T7 RNA polymerase and template DNA [PCR products with sequence-specific primers (cr5.2PCRf/r or cr5.13PCRf/r, respectively)] at 37°C for 4 h. For the self-cleaving reaction of the hammerhead ribozyme, the transcription reaction was directly diluted with 4 volumes of 30 mM MgCl_2_ in DEPC-H_2_O and incubated for 1 h at 60°C. The cleaved crRNA was purified by phenol/chloroform extraction (pH 5.2), EtOH precipitated with the addition of glycogen (1:100, v/v), mixed with 2× formamide loading buffer (95% formamide, 5 mM EDTA, pH 8.0, 2.5 mg bromophenol blue, 2.5 mg xylene cyanol), heated for 5 min at 95°C, separated by a denaturing-PAGE (8 M urea, 1× TBE, 10% polyacrylamide) next to an RNA marker (low range ssRNA ladder, NEB) and visualized by toluidine blue staining. The gel bands were cut out and eluted overnight on ice in 500 µl elution buffer (20 mM Tris–HCl, pH 7.5, 250 mM sodium acetate, 1 mM EDTA, pH 8.0, 0.25% SDS) and EtOH precipitated. All DNA oligonucleotides used for cloning and as cleavage substrates were custom-synthesized (Eurofins MWG Operon, Supplementary Table SI).

### RNA sequencing

The isolated small RNA was treated with T4 Polynucleotide Kinase (PNK) to ensure proper termini for adapter ligation (45). First, for the dephosphorylation of 2′–3′-cyclic phosphate termini, 10 µg of RNA was incubated at 37°C for 6 h with 20 U T4 PNK (NEB) in 1× T4 PNK buffer. Subsequently, 1 mM ATP was added, and the reaction mixture was incubated for 1 h at 37°C to generate monophosphorylated 5′-termini (46). RNA libraries were prepared with an Illumina TruSeq RNA Sample Prep Kit (Ambion), and sequencing on an Illumina HiSeq2000 sequencer was performed at the Max-Planck Genome Centre, Cologne, Germany. Reads were mapped to the *T. tenax* reference genome (FN869859) with CLC Genomics Workbench 6.0.

### Purification of Cascade proteins

The gene constructs of *csa5*, *cas7*, *cas5a*, *cas3*′, *cas3*′′ and *cas8a2* in pET24a(+) (Novagen) were used as previously described (41). Cas3′′ mutants were created using the QuikChange site-directed mutagenesis protocol (Stratagene) according to the manufacturer's instructions. Established mutations were confirmed by sequencing (MWG Eurofins). Soluble Csa5 could be purified, as cells were homogenized in buffer 1 (100 mM HEPES/KOH, pH 7, 10% glycerol, 10 mM ß-mercaptoethanol (ß-Me), 10 mM CaCl_2_, 300 mM NaCl), lysed, cleared by centrifugation (45 000 × *g*, 1 h, 4°C) and heat precipitated (30 min, 90°C). The cleared supernatant (14 000 × *g*, 30 min, 4°C) was dialyzed overnight in salt-free lysis buffer 1 and purified via a Hi Screen Blue FF affinity column using a FPLC Äkta-Purification system (GE Healthcare) eluted with a linear salt gradient (0–2 M NaCl) at 420 mM NaCl. Fractions containing Csa5 were pooled, dialyzed overnight in salt-free buffer 1 and purified via an anion-exchange chromatography with a MonoQ 5/50 GL column (GE Healthcare) eluted with a linear salt gradient (0–1 M NaCl) at 100 mM NaCl. For pull-down assays of Cascade *in vivo*, Csa5 with a C-terminal 6× His-tag was used as a bait protein. Therefore, *csa5* was cloned into pET20b(+), protein expressed and cells lysed in buffer 1 without CaCl_2_. The Csa5-His protein was purified from *E. coli* cell lysate by Ni-NTA affinity chromatography (HisTrap HP, GE Healthcare) and eluted with a linear imidazole gradient (0–500 mM) at 150 mM imidazole. Cas7 was purified by cell lysis in buffer 1, heat precipitation at 80°C and cation exchange chromatography with Heparin Sepharose (GE Healthcare) as described earlier (41). Additionally, the Cas7 full-length protein was expressed as a recombinant His-SUMO-tagged fusion protein using BL21-Gold(DE3)Star pRARE (Stratagene) overnight at 18°C. The cells were lysed by sonication in buffer 2 (100 mM potassium phosphate, pH 7.5, 500 mM NaCl, 10% glycerol, 1 mM ß-Me) supplemented with 20 mM imidazole, benzonase (NEB) and protease inhibitors (Roche), and Cas7-SUMO was further purified using Ni-NTA affinity chromatography. The salt concentration was lowered to 100 mM NaCl in 50 mM Tris, pH 7.5, and the His-SUMO-tag was cleaved off by adding SUMO protease overnight during dialysis. The protein was further purified over a HiTrap Heparin Sepharose HP column (GE Healthcare) to remove non-specifically bound nucleic acids. Size-exclusion chromatography on a Superdex 75 column (GE Healthcare) was preformed as a final step of purification in buffer 3 (100 mM NaCl, 50 mM Tris, pH 7.5, 10% glycerol, 5 mM dithiothreitol). The insoluble proteins Cas5a, Cas3′, Cas3′′ and Cas8a2 were purified from inclusion bodies and solubilized in 4 M guanidine hydrochloride (GdmCl). The purity of all proteins was determined by sodium dodecyl sulfate-polyacrylamide gel electrophoresis (SDS–PAGE) and Coomassie blue staining alongside the protein marker (ColorPlus™ prestained protein ladder, broad range, NEB). The protein concentration was determined by the Bradford protein quantification method (BioRad).

### Reconstitution assays

The reconstitution of the Cascade complex was previously described (41). Briefly, equal amounts (300 µg) of each solubilized protein Cas5a, Cas3′, Cas3′′ and Cas8a2 were mixed with the purified proteins Csa5 and Cas7 in reconstitution buffer (3.5 M GdmCl, 100 mM HEPES/KOH, pH 7, 5% glycerol, 10 mM ß-Me, 10 mM MgCl_2_, 300 mM NaCl) and the denaturing agent was removed via stepwise dialysis into GdmCl-free buffer at room temperature. Aggregated proteins were precipitated (14 000 × *g*, 30 min, 4°C), soluble proteins concentrated with centrifugal filter units (MWCO: 10 kDa) and analyzed by SDS–PAGE. The reconstitution of all 63 subunit combinations was tested via rapid dilution into 1 ml GdmCl-free reconstitution buffer of equal amounts (25 µg) of each subunit. To minimize aggregation, the protein solution was added drop-wise (5 µg total protein) to the reconstitution buffer. Aggregated proteins were precipitated (14 000 × *g*, 30 min, 4°C), soluble proteins were trichloroacetic acid (TCA)-precipitated and the identical amounts of supernatant and pellet analyzed by SDS–PAGE.

### Pull-down assays and mass spectrometry analyses of *in vivo* Cascade proteins

To determine the protein–protein interaction *in vivo*, pull-down assays were performed with C-terminal His-tagged Csa5 used as a bait protein bound to a cobalt-chelate matrix and *T. tenax* cell extract as a prey. For the production of cell-free *T. tenax* extract, 1 g cells were resuspended in lysis buffer 3 (100 mM HEPES/KOH, pH 7.5, 300 mM ß-Me) and lysed as described elsewhere (47). To stabilize the interaction of proteins, a chemical cross-linking with formaldehyde was performed. One hundred micrograms purified Csa5, 1 mg cell extract and 1% formaldehyde (37% formaldehyde/10% MeOH) were mixed, incubated for 15 min on ice and the reaction stopped by the addition of 200 mM glycine for 5 min on ice. Subsequently, the pull-down assay was performed according to the ProFound Pull-Down PolyHis Protein:Protein Interaction Kit (Pierce). The cross-linked protein:cell extract was incubated for 30 min at 4°C on the cobalt resin, washed five times with 40 mM imidazole in buffer 1 without CaCl_2_ and eluted at 290 mM imidazole. Proteins were TCA-precipitated, separated by SDS–PAGE and visualized with silver staining (Pierce Silver Stain Kit). For the mass spectrometry analysis, the protein:cell extract was pelleted by EtOH precipitation and digested in solution in the presence of urea as described previously (48). The resultant peptides were desalted using STAGE tips (49). Further, the desalted peptides were analyzed by liquid chromatography tandem mass spectrometry on an LTQ Orbitrap Velos instrument (Thermo Fischer Scientific) under standard conditions. The protein identification was performed with MaxQuant (version 1.2.2.5) using the Andromeda search engine (50,51).

### Protein–protein and protein–RNA interaction assays

The protein–protein interaction and the native molecular mass of Cascade or the Cas7 subunit was determined by size-exclusion chromatography with a Superdex 200 10/300 preparatory-grade column (GE Healthcare, 24 ml). Protein containing fractions were TCA-precipitated [1:4 of 100% (v/v)] and analyzed by SDS–PAGE. To verify the binding of Cascade to crRNA, 50 µg of synthetic crRNA was added to 500 µg of reconstituted Cascade complex incubated at 65°C for 30 min to facilitate RNA binding and separated by gel filtration. Fractions were split and protein extracted via TCA precipitation and analyzed during separation on SDS–PAGE, whereas RNA was isolated by Proteinase K treatment (10 µl, 6 U Proteinase K, 30 min at 37°C), phenol/chloroform extraction, EtOH precipitation and detection via denaturing-PAGE (10% polyacrylamide) and toluidine blue staining.

### Electrophorectic mobility shift assay

The Cascade complex or the Cas7 subunit were tested for the ability of binding synthetic crRNA in electrophorectic mobility shift assays (EMSAs). As the binding of Cascade was metal-independent, the complex was reconstituted in the presence of 10 mM CaCl_2_ instead of MgCl_2_ to reduce RNA cleavage. A total of 5 pmol of the synthetic crRNA substrate was 5′-labeled with [γ-^32^P]-ATP (5000 ci/mmol, Hartmann Analytic) and T4 PNK (Ambion) for 2 h at 37°C; the reaction stopped by the addition of formamide loading buffer and the separation by denaturing-PAGE (10% polyacrylamide). After autoradiographic exposure, the RNA band were cut out, gel eluted and EtOH precipitated. Up to 5 µM of reconstituted Cascade was incubated with 2–4 nM of 5′-labeled crRNA in binding buffer (100 mM HEPES/KOH, pH 7, 100 mM NaCl, 10 mM ß-Me, 50 ng yeast RNA) for 30 min at 70°C. The reaction was immediately stopped on ice, mixed with 1× loading buffer (Qiagen) and Cascade:crRNA complexes separated from free crRNA by non-denaturing TBE-PAGE (6% polyacrylamide, 1× TBE). The ability of monomeric Cas7 to bind to synthetic crRNA was tested by incubating protein with 2–4 nM of 5′-labeled crRNA in binding buffer without yeast RNA for 30 min at 80°C, ran on 2% TAE-agarose gels and visualized by phosphorimaging. Binding of the two Cas7 versions (Cas7, Cas7-SUMO) to contaminating nucleic acids during the purification process was verified by loading 100 µg protein on 1% TAE-agarose gels stained with ethidium bromide alongside the DNA marker (2-log DNA ladder, NEB). The specification of the contaminated nucleic acid was conducted by adding 2 U DNase I (RNase-free, NEB), 1 ng RNase A (biochemistry grade, Ambion) or 25 U benzonase (NEB) in the appropriate reaction buffer to the protein solution and incubated for 30 min at 37°C before loading on agarose gels.

### Transmission electron microscopy (TEM)

To further analyze samples of Cas7 purifications, 5 µl of a concentrated protein solution was applied to carbon-coated 400 mesh copper grids. After blotting on a filter paper, the samples were washed twice on a drop of double distilled water and blotted each time. Finally, the proteins were negatively stained with 2% (w/v) uranyl acetate for 20 s, blotted again on filter paper and left for air drying (52,53). Electron microscopy and further analysis was carried out on a JEOL JEM-2100 transmission electron microscope (JEOL, Tokyo, Japan) operated at 120 kV. The microscope was equipped with a 2k × 2k CCD camera F214 in combination with the EM-Menu 4 software (TVIPS, Gauting, Germany).

### Nuclease assays

The reconstituted Cascade was tested for the nucleolytical activity of the Cas3′′ subunit. A total of 5 pmol of the ssDNA oligonucleotide (int 5.2_CCT for) was 5′-labeled with [γ-^32^P]-ATP (5000 ci/mmol, Hartmann Analytic) and purified as described earlier in the text. In all, 100 nM refolded Cascade was incubated with 2 nM of 5′-labelled ssDNA in nuclease buffer (100 mM HEPES/KOH, pH 7.0, 100 mM NaCl, 5 mM MgCl_2_, 5 mM MnCl_2_, 10 mM ß-Me, 20 ng yeast total RNA) at 70°C for 10 min, the reaction stopped via EtOH precipitation and resuspended in 10 mM Tris–HCl, pH 8.5, and formamide loading buffer. Each sample was boiled, measured via scintillation counting (Beckman LS6000) and samples with identical counts per minute after normalization were loaded on a 15% denaturing polyacrylamide gel alongside the low molecular weight marker (10–100 nt, Affymetrix) running in 1× TBE (8 watts, 2 hours). Cleavage products were visualized by phosphorimaging.

To test for the cleavage of dsDNA, short substrates were designed that mimic an invasive viral DNA. Therefore, the spacer sequence of crRNA 5.2 or 5.13 was used, the different PAM added upstream and flanked with random sequences up- and downstream (T7 promoter and T7 terminator, respectively) and each DNA oligonucleotide was synthesized either in forward or reverse complementary direction (for/rev: int 5.2_CCT, int 5.2_CCA, int 5.2_TCA, int 5.2_TCG, int 5.2_AAA, int 5.2_Rep or int 5.13_CCT, respectively). A total of 5 pmol of each forward and reverse oligonucleotide was 5′-labeled with [γ-^32^P]-ATP (5000 ci/mmol, Hartmann Analytic), purified as mentioned earlier and hybridized with 1.5-fold molar excess of the respective cold complementary strand by heating at 95°C for 5 min and slowly cooling down to room temperature in hybridization buffer (10 mM Tris–HCl, pH 8, 1 mM EDTA, pH 8, 100 mM NaCl). For interference tests, 500 nM refolded Cascade and 500 nM crRNA (crRNA 5.2 or 5.13) were incubated in interference buffer 1 (100 mM HEPES/KOH, pH 7.0, 100 mM NaCl, 10 mM ß-Me, 20 ng yeast total RNA) at 70°C for 20 min to allow loading of crRNA into Cascade and then the reaction started by adding 2 nM of 5′-labeled hybridized dsDNA and interference buffer 2 (5 mM MgCl_2_, 5 mM MnCl_2_, 2 mM ATP) at 70°C for 10 min. The reaction was stopped via EtOH precipitation and loaded on 20% denaturing TBE-polyacrylamide gels or on 10% sequencing gels (Model S2 sequencing gel electrophoresis apparatus, Life Technologies) alongside the low molecular weight marker or (10–100 nt) or seven specific labeled fragments (8–66 nt). 

## RESULTS

### RNA-Seq analyses reveal the processing pattern of *T. tenax* crRNAs

**Figure 1.**
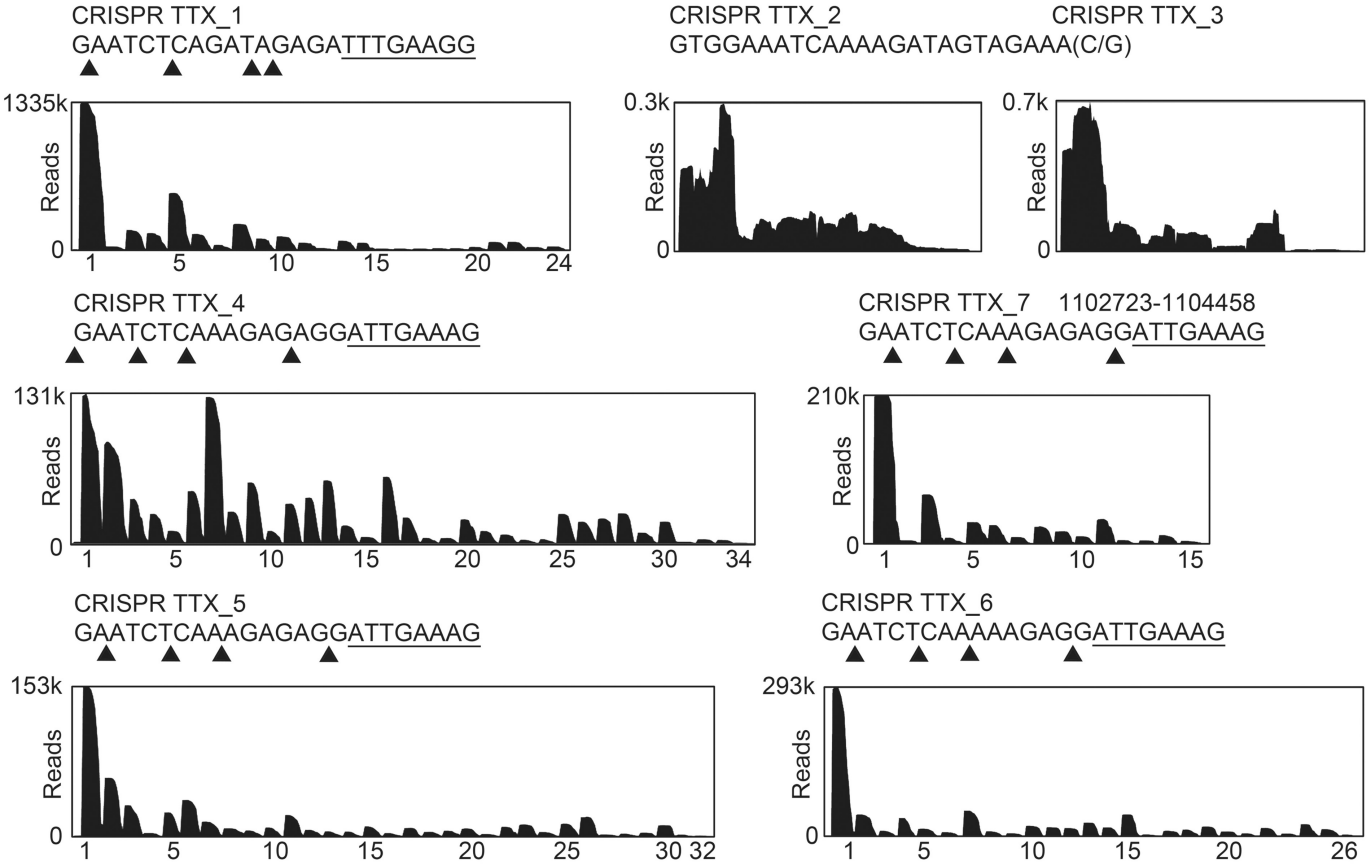
Processing of crRNAs in *T. tenax*. The abundance of crRNAs was verified by mapping the Illumina HiSeq2000 sequencing reads to the *T. tenax* Kra1 reference genome. Sequence coverage (reads) for the seven *T. tenax* CRISPR clusters (crRNAs are indicated by numbers on the *x*-axis) is visualized. The processing sites could be identified within the repeat elements, generating crRNAs with a 5′-terminal 8-nt tag [5′-(A/U)UUGAA(A/G)G-3′, underlined] and variable trimming of the 3′-ends with most of the crRNAs terminating with a 1–2-nt tag (5′-G/GA-3′). The trimming sites for each CRISPR locus are indicated by triangles.

One requirement for CRISPR immunity is the processing of crRNA precursors to yield mature crRNAs. To obtain a comprehensive view of the processing pattern of crRNAs in *T. tenax*, total small RNA was isolated and sequenced via Illumina HiSeq2000 RNA-Seq methodology. T4 PNK treatment of *T. tenax* small RNAs was required to facilitate adaptor ligation during RNA-Seq library construction, which confirms the presence of 5′-OH termini (45). Previously, seven CRISPR loci with a total of 142 spacer sequences were identified, and crRNA transcripts were verified for five clusters (TTX_1, 4, 5–7) via northern blot detection, whereas two clusters (TTX_2–3) showed no distinct processing pattern (41). In total, 13 357 720 individual sequence reads were mapped to the *T. tenax* reference genome (FN869859). Constitutive crRNA production and a gradual decline of mature crRNAs from the leader proximal to the leader distant region within each active cluster were identified (Figure 1). The read coverage indicates the relative abundance and the processed termini of crRNAs from the different CRISPR arrays. Individual crRNAs for the first spacers were represented by up to 1 334 585 reads (spacer 1.1, 10% of total reads), which demonstrates a massive accumulation of crRNAs *in vivo*. All identified crRNAs contained a clearly defined 5′-terminal 8 nt tag (TTX_1: 5′-UUUGAAGG-3′ or TTX_4–7: 5′-AUUGAAAG-3′). The 3′-termini are gradually shortened and mostly contain a minimal 2-nt tag (5′-GA-3′). Additionally, two spacers (spacer 4.34 and spacer 5.32) that were not listed in the CRISPR database could be identified, increasing the total number of 144 spacers in *T. tenax*. In contrast, the CRISPR loci (TTX_2–3) were inactive and showed no mature crRNAs with defined termini.

### Cas3′ and Cas3′′ are an integral part of the type I-A Cascade complex

Typical for archaeal subtype I-A CRISPR systems is the organization of Cascade genes in an operon structure and their transcription as a polycistronic unit, which includes individual genes for the helicase (Cas3′) and nuclease (Cas3′′) domains of Cas3 (41). This organization suggests a different complex formation than Cascade:Cas3 complexes of the bacterial subtype I-E, in which Cas3 is recruited to an assembled Cascade bound to target DNA. To substantiate the protein–protein interactions of the Cascade complex *in vivo*, we performed pull-down assays of *T. tenax* cell extracts with the C-terminal His-tagged Csa5 as bait protein (Supplementary Figure S1A). The interacting proteins were subjected to an in solution trypsin digest followed by mass spectrometry analyses. In these experiments, we could identify Cas7, Cas5a, Cas3′, Cas3′′ and Cas8a2 as interaction partners of Csa5, all six proteins whose genes are organized in an operon (Supplementary Table SII). This suggests an alternative mechanism of Cascade formation, in which the two Cas3 domains are not recruited but are an integral part of the Cascade complex in advance of the immune response. Noteworthy, none of the two copies of Cas6 in the genome of *T. tenax* were found in the data. Additionally, we could also detect a second Cas7 homolog (TTX_0235, 40% similarity), produced from a distant *cas* gene cluster without an encoded *csa5* gene, as a potential interaction partner of Csa5.

### The *in vitro* assembled Cascade efficiently binds a synthetic mature crRNA

**Figure 2.**
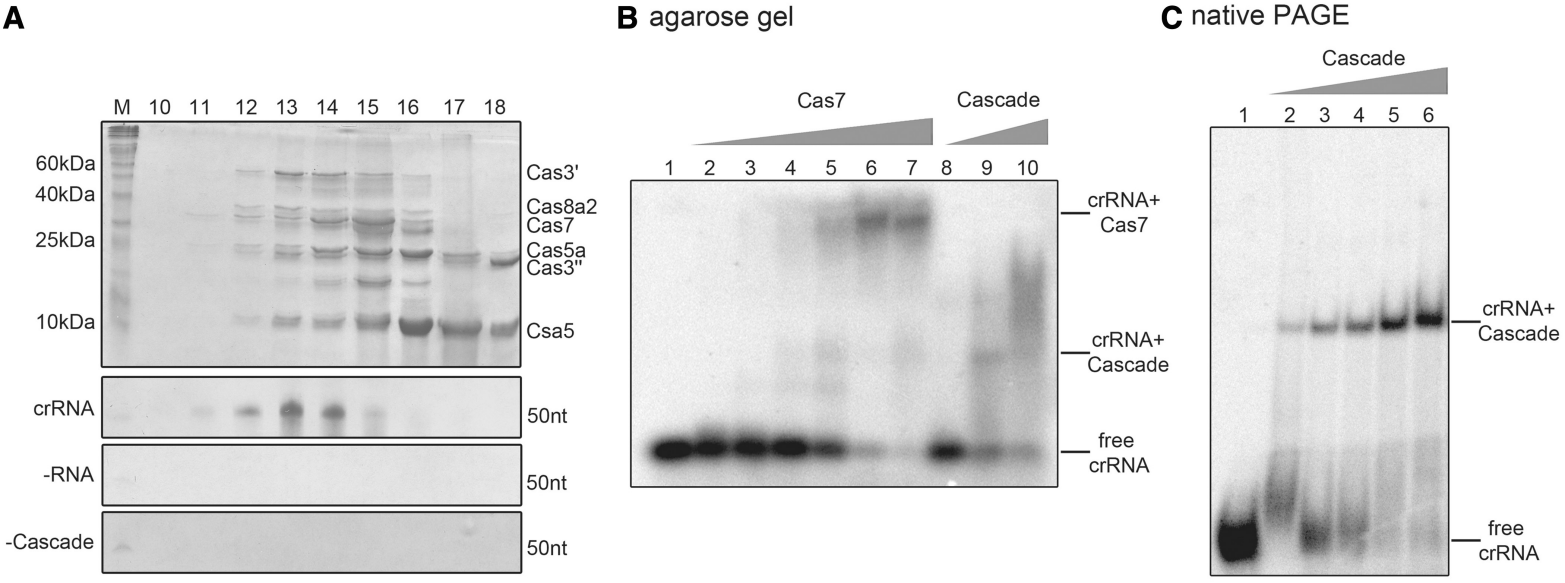
Cascade assembly and RNA binding. (**A**) The Cascade subunits (Csa5, Cas7, Cas5a, Cas3′, Cas3′′ and Cas8a2) were assembled via a co-refolding procedure of insoluble recombinant proteins, incubated with synthetic crRNA 5.2 (crRNA) or no RNA (−RNA), and protein interaction was verified via size-exclusion chromatography. In all, 15% SDS–PAGE of individual fractions (fractions 10–18) shows distinct protein bands of assembled Cas protein subunits. The Cascade-containing fractions were additionally analyzed for bound RNA content by 10% Urea-PAGE. A protein-free sample served as a running control (−Cascade). (**B**) A comparison of the EMSAs for crRNA binding by the individual Cas7 subunit (lanes 1–7: 0, 0.2, 0.5, 1, 2, 4, 5 µM) or Cas7 assembled into Cascade (lanes 8–10: 0.5, 2, 5 µM) on 1% agarose gels demonstrates the formation of high shifts for Cas7 alone and lower, more diffuse shifts for Cascade. (**C**) EMSAs verified the binding of the 5′-[γ-^32^P]-ATP labeled crRNA 5.13 by Cascade (with Cas7 purified via a SUMO-fused construct) in increasing concentrations (lanes 1–6: 0, 0.125, 0.25, 0.5, 1, 2 µM) and in the presence of yeast RNA via 6% native PAGE.

To obtain mature crRNAs with an 8 nt tag (5′-AUUGAAAG-3′) and a 5′-hydroxyl-terminus, we designed a ribozyme maturation strategy that overcomes the need for recombinant Cas6 to be available. A DNA construct was designed that contained a minimal *cis*-acting hammerhead ribozyme fused to the sequence of a mature crRNA under the control of a T7 RNA polymerase promoter (Supplementary Figure S2A). The synthetic crRNA was obtained by *in vitro* transcription and self-cleavage of the RNA transcript at 60°C. This methodology was tested for two different crRNA constructs [crRNA 5.2 (50 nt) and 5.13 (54 nt)], resulting in a cleavage efficiency of ∼60% (Supplementary Figure S2B) (54,55). These synthetic mature crRNAs were tested for their capability in complex binding to establish a fully *in vitro* assembly strategy of functional Cascade modules. In this procedure, the co-refolding of the insoluble subunits Cas5a, Cas3′, Cas3′′ and Cas8a2, in combination with the purified proteins Csa5 and Cas7, yielded soluble Cascade (Supplementary Figure S1A–C). First attempts showed an increased refolding efficiency in the combination of all six Cascade subunits (41). Therefore, we investigated the co-refolding of all possible 63 compositions of Cascade subunits to scan for a minimal stable core of Cas proteins. Surprisingly, only the co-refolding of all six proteins resulted in high amounts of soluble protein recovery of ∼50%, whereas in contrast all other 62 combinations resulted in negligible amounts (up to 10%) of soluble protein (Supplementary Figure S3). An up-scaled protocol for the assembly of the six-protein Cascade revealed soluble proteins with concentrations of up to 4 mg/ml. The assembled Cascade was then incubated for 30 min at 65°C to remove impurities or misfolded heat-instable subunits before size-exclusion chromatography for screening of Cascade complex formation. The analysis of the elution profile (12–17 ml elution volume) showed that all six co-refolded proteins eluted in one fraction, followed by smaller sub-complexes and monomeric protein subunits (Figure 2A and Supplementary Figure S4). The functionality of Cascade was first assayed by analysis of binding to the synthetic crRNA. The assembled complex was incubated with crRNA, applied to size-exclusion chromatography and identical fractions were checked for protein and RNA content. The first fractions of the Cascade profile co-eluted with the added crRNA. Visible on the denaturing gel are bands at the expected size of ∼50 nt (Figure 2A, crRNA). Two controls showed that the assembled Cascade was not cross-contaminated with small RNA from the *E. coli* expression host (Figure 2A, −RNA) and unbound crRNA was not eluting in the respective fractions (Figure 2A, −Cascade). Thus, mature crRNAs are supporting the Cascade formation, and the established ribozyme methodology can be used to prepare synthetic crRNA molecules to be loaded onto Cascade.

In type I-E systems, the subunit Cas7 constitutes the helical backbone of Cascade that is formed on binding of the crRNA (31). To verify the RNA-binding activity of Cas7 in I-A systems, we analyzed *T. tenax* Cas7 in more detail. The purification strategy for untagged soluble Cas7 resulted in multimeric proteins that eluted at the void volume from the used gel filtration column indicating protein complex sizes >600 kDa (Supplementary Figure S5A). The treatment of Cas7 with DNase I, RNase A or benzonase identified a cross-contamination of Cas7 with *E. coli* RNAs (<500 nt), which suggests that Cas7 multimerization is obtained by RNA binding independent of its sequence (Supplementary Figure S5B). Transmission electron microscopy (TEM) of this sample revealed long helical filaments of up to 50-nm length, similar to previously reported structural studies of *S. solfataricus* (43). Additionally, we could also observe the occurrence of interlaced filaments of two Cas7 helices illustrating the potential for complete coverage of RNA molecules with Cas7 subunits (Supplementary Figure S6).

To prevent polymeric Cas7 artifacts, an alternative purification strategy of a Cas7-SUMO fusion protein was established that resulted in monomeric and RNA-free Cas7 (Supplementary Figure S5A and C). This protein preparation was subsequently used for the *in vitro* assembly of Cascade (Figure 2A). A comparison of the crRNA binding behavior in EMSAs showed the recurrence of high-shifting multimeric Cas7 structures for the sole Cas7 protein (Figure 2B) and lower shifts and an increased affinity toward crRNA for Cas7 in a Cascade assembly. The addition of total yeast RNA or unlabeled crRNA resulted in similar Cas7 band shifts and underlines an unspecific affinity toward RNA for the individual Cas7 subunit (Supplementary Figure S7). In contrast, specific binding of Cascade to the synthetic crRNA 5.13 was seen in EMSAs, with protein concentrations ranging from 0.125–2 µM (Figure 2C). A second synthetic crRNA (crRNA 5.2) is bound by Cascade with a similar affinity (Supplementary Figure S8). 

### The exonucleolytically ssDNA cleavage activity of Cas3′′ is inhibited by the addition of crRNAs

**Figure 3.**
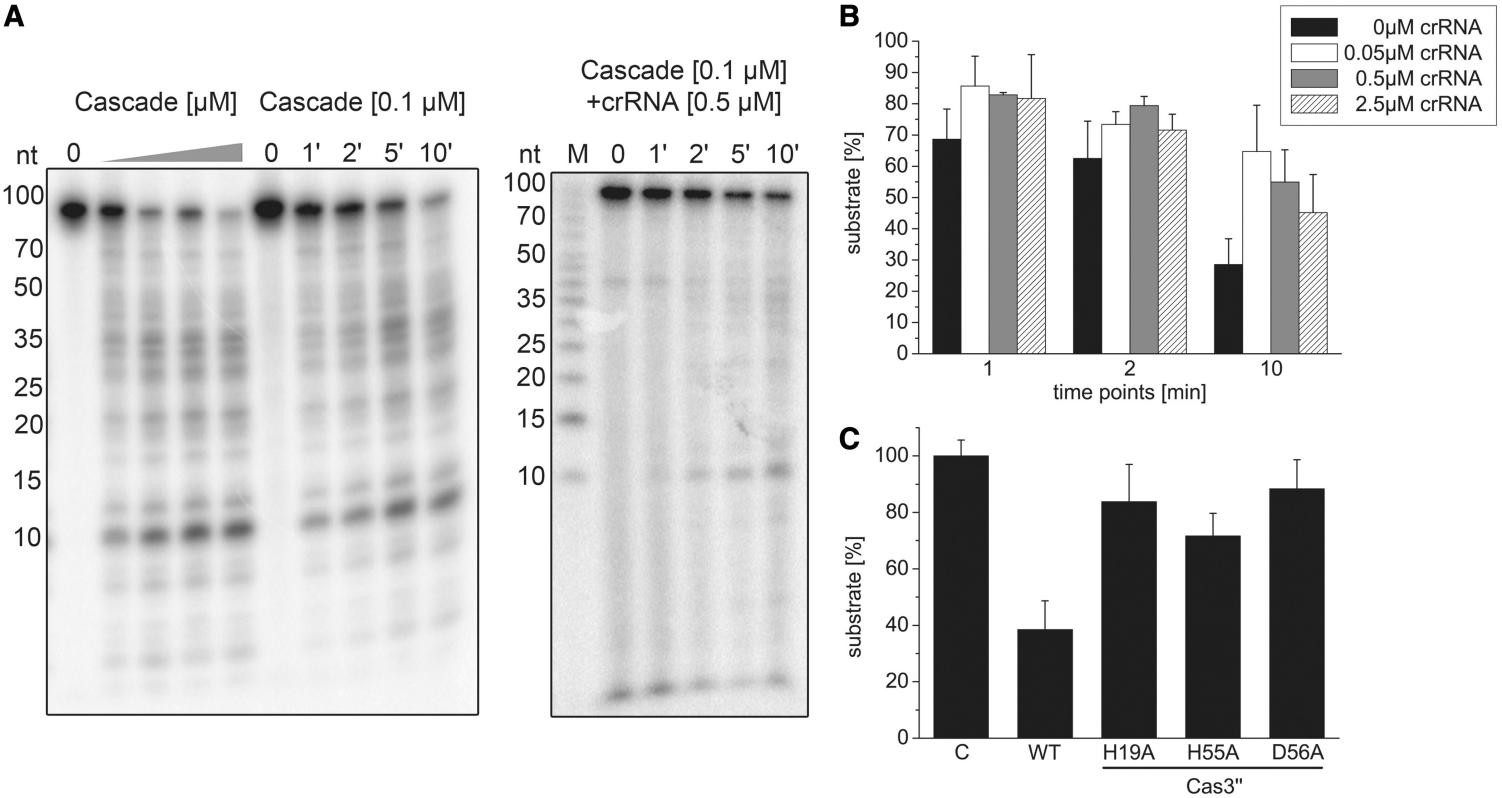
Type I-A Cascade cleaves ssDNA unspecifically and is inhibited by crRNA. (**A**) Cascade (0, 0.05, 0.125, 0.25, 0.5 µM) was incubated with a 5′-[γ-^32^P]-ATP labeled ssDNA fragment (int_5.2 CCT for) in the presence of Mg^2+^ and Mn^2+^ ions and the nucleolytic cleavage reaction was resolved on 15% denaturing gels. After 1–10-min incubation at a fixed Cascade concentration of 0.1 µM, >70% of the substrate is cleaved. In the presence of 0.5 µM unlabeled crRNA, the reaction is inhibited. (**B**) This observation was tested in the presence of 0, 0.05, 0.5 and 2.5 µM crRNA, and the amount of remaining substrate estimated via line profile plots (Image J) was plotted for three different reaction times (1, 2, 10 min) and reactions performed in triplicate. (**C**) Cas3′′ subunit with HD domain mutations (H19A, H55A, D56A) was assembled into Cascade and then tested in ssDNA cleavage assays.

Previous analysis of the nuclease domain of a Cas3 enzyme indicated endo- and exonucleolytic ssDNA cleavage and revealed an HD motif within its active site, which is involved in the coordination of transition metal ions (38). Therefore, nuclease assays with a 93 nt linear ssDNA substrate were performed to biochemically characterize the *T. tenax* Cas3′′ subunit within a coordinated Cascade structure. Nuclease assays with an assembled Cascade lacking crRNA showed increasing cleavage activity at Cascade concentrations of 0.05–0.5 µM. At the highest Cascade concentration, up to 75% ssDNA was degraded during 10 min incubation at 70°C. A time course cleavage assay with 0.1 µM Cascade demonstrated degradation of the substrate within 10 min (Figure 3A). The cleavage reaction showed a strict dependence on divalent metal ions, as increased cleavage of Cascade was only observed in the presence of Mn^2+^ ions, followed by Mg^2+^ ions, while Ca^2+^ ions inhibited the reaction. The highest cleavage rate was observed with the combination of Mg^2+^ and Mn^2+^ ions (Supplementary Figure S9). Next, we tested the influence of crRNA addition on the unspecific ssDNA nuclease activity. The used crRNA (crRNA 5.2) and the ssDNA fragment (int 5.2_CCT for) are not complementary to each other to prevent the formation of non-cleavable RNA:DNA hybrids. Cascade was first loaded with crRNA, followed by the addition of the ssDNA substrate to the reaction to mimic the *in vivo* situation of Cascade assembly. The comparison of Cascade ssDNA cleavage rate at 0.1 µM without crRNA and with 0.5 µM crRNA for the identical time points showed an inhibition of the nuclease activity (Figure 3A). The strongest inhibitory effect could be observed at 0.05 µM added crRNA after 10 min incubation, which resulted in an over twofold decrease in activity (65% versus 28.5% remaining ssDNA). The lowered cleavage rate can also be seen for higher concentrations of crRNA (0.5 µM: 55%, 2.5 µM: 45% uncleaved substrate), but is less significant, presumably due to hybrid formation of excess crRNAs (Figure 3B). To verify that Cas3′′ is the sole ssDNA nuclease domain within the archaeal Cascade, we aligned the *T. tenax* Cas3′′ with the previously characterized *Methanocaldococcus jannaschii* Cas3′′ (MJ0384) sequence and introduced mutations of three of the four highly conserved residues of the HD domain motif (H19A, H55A and D56A) that are expected to inactivate exonuclease cleavage (37). The produced Cas3′′ mutants were isolated and purified according to the wild-type Cas3′′ purification protocol and assembled within Cascade via the co-refolding procedure (Supplementary Figure S10). Each Cas3′′ mutant showed a deficiency in ssDNA nuclease activity, most dramatically observed for D56A (12% cleaved ssDNA), followed by H19A (18% cleaved substrate) (Figure 3C).

### Cascade-mediated interference is dependent on crRNA and the protospacer DNA

**Figure 4.**
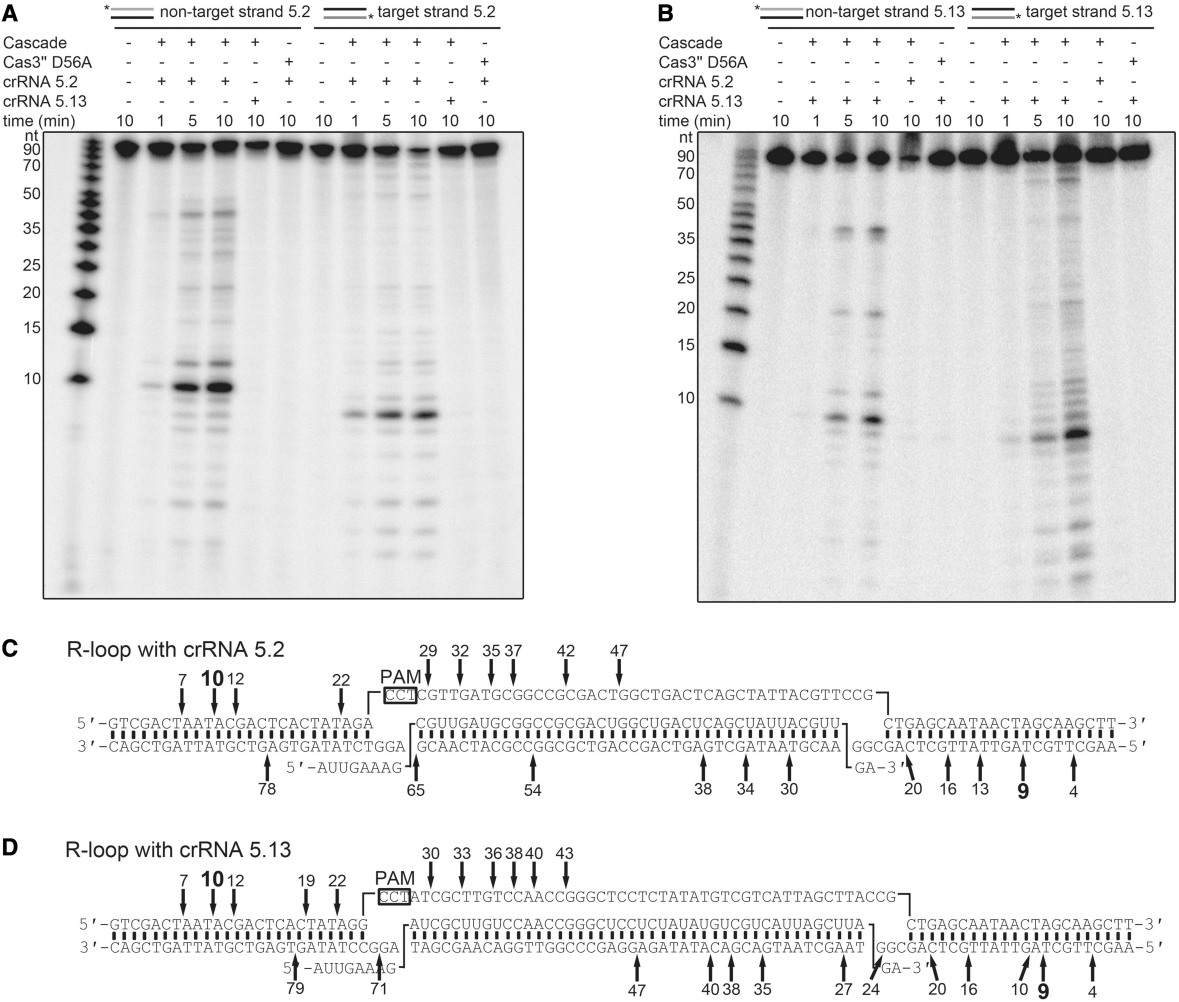
Interference activity of *in vitro* assembled type I-A Cascade. (**A**) The assembled Cascade complex is loaded with crRNA 5.2 for 20 min at 70°C, and the interference reaction is started with the addition of ATP, Mg^2+^, Mn^2+^ and the dsDNA substrate (in_5.2 CCT), which is either 5′-[γ-^32^P]-ATP labeled on the non-target (forward) or the crRNA target strand (reverse). Cleavage reactions were stopped at three different time points (1, 5, 10 min at 70°C). The reaction products of the cleaved dsDNA were separated on 20% denaturing gels. The non-matching crRNA 5.13 and the Cas3′′ D56A mutant are used as controls. (**B**) In parallel, the crRNA 5.13 is loaded into Cascade, and cleavage of the matching dsDNA substrate (in_5.13 CCT) is visualized. (**C** and **D**) The cleavage products are analyzed on 10% Urea-PAGE for each strand (in_5.2 CCT for/rev and in_5.13 CCT for/rev) with two different markers (Supplementary Figure S9). The cleavage sites are marked within the proposed R-loop structure that is formed during the interference reaction [(C) dsDNA 5.2, (D) dsDNA 5.13].

Next, the assembled I-A Cascade was tested for cleavage of dsDNA in dependence of the spacer encoded crRNA to show *in vitro* type I-A Cascade-mediated interference. First, short dsDNA fragments were designed that mimic viral protospacer DNA. The spacer sequence 5.2 (40 bp) was flanked by random sequences on both sides (25 bp each). Directly upstream of the spacer the PAM sequence CCT was integrated. The PAM sequence CCN was identified in *S. solfataricus* on the basis of viral BLAST hits of spacer targets (10). The non-target (int 5.2_CCT for, crRNA non-complementarity) or the target DNA strand (int 5.2_CCT rev, crRNA complementarity) was 5′-labeled and hybridized with 1.5-fold excess of the complementary cold strand to obtain two labeled dsDNA fragments. The synthetic mature crRNA construct 5.2 was supplied by hammerhead self-cleavage and first loaded into Cascade before the addition of the dsDNA substrates, the metal ions Mg^2+^ and Mn^2+^, as well as ATP. The separation of cleavage products via 20% denaturing-PAGE identified a Cascade-dependent degradation pattern of the dsDNA substrates with differences in fragment length for the non-target and target strand (Figure 4A). As a control, the Cas3′′ nuclease mutant D56A was assembled into Cascade, which showed no cleavage activity of the protospacer DNA. Cascade loaded with a synthetic crRNA 5.13 that is not complementary to the DNA target showed also no dsDNA cleavage activity demonstrating crRNA-mediated target guidance. In return, the reaction was performed using the spacer sequence 5.13 with identical flanks in the dsDNA substrate as used before (Figure 4B). Accordingly, only the matching crRNA 5.13 was capable to guide the Cascade-mediated cleavage of the dsDNA substrate, whereas neither the incorporated crRNA 5.2 nor the Cascade mutant Cas3′′ D56A showed nuclease activity. The cleavage pattern of the non-target and target strand was similar for dsDNA 5.13 in comparison with dsDNA 5.2. Therefore, the cleavage products of the interference reaction with the labeled non-target and target strand of each dsDNA substrate 5.2 or 5.13 were separated on 10% sequencing gels to specify the fragment lengths (Supplementary Figure S11). Each reaction was loaded next to a marker with ssDNA fragments of 10–100 nt length and a mixture of seven ssDNA fragments of 8–66 nt length to pinpoint the dominant cleavage products. Cleavage sites were observed in the middle of the non-target strand within the protospacer (at position 43–47 nt), and smaller 5′-terminal fragments were generated with a minimal length of 10 nt (Figure 4C). The target strand is cleaved over its entire length, with smallest fragments of 9 nt. The comparison of both DNA substrates showed no significant differences in the fragmentation pattern, indicating a sequence unspecific cleavage of Cascade in dependence of the position within the R-loop structure (Figure 4D).

### The Cascade reconstitution platform allows the identification of PAM-dependent target DNA cleavage

**Figure 5.**
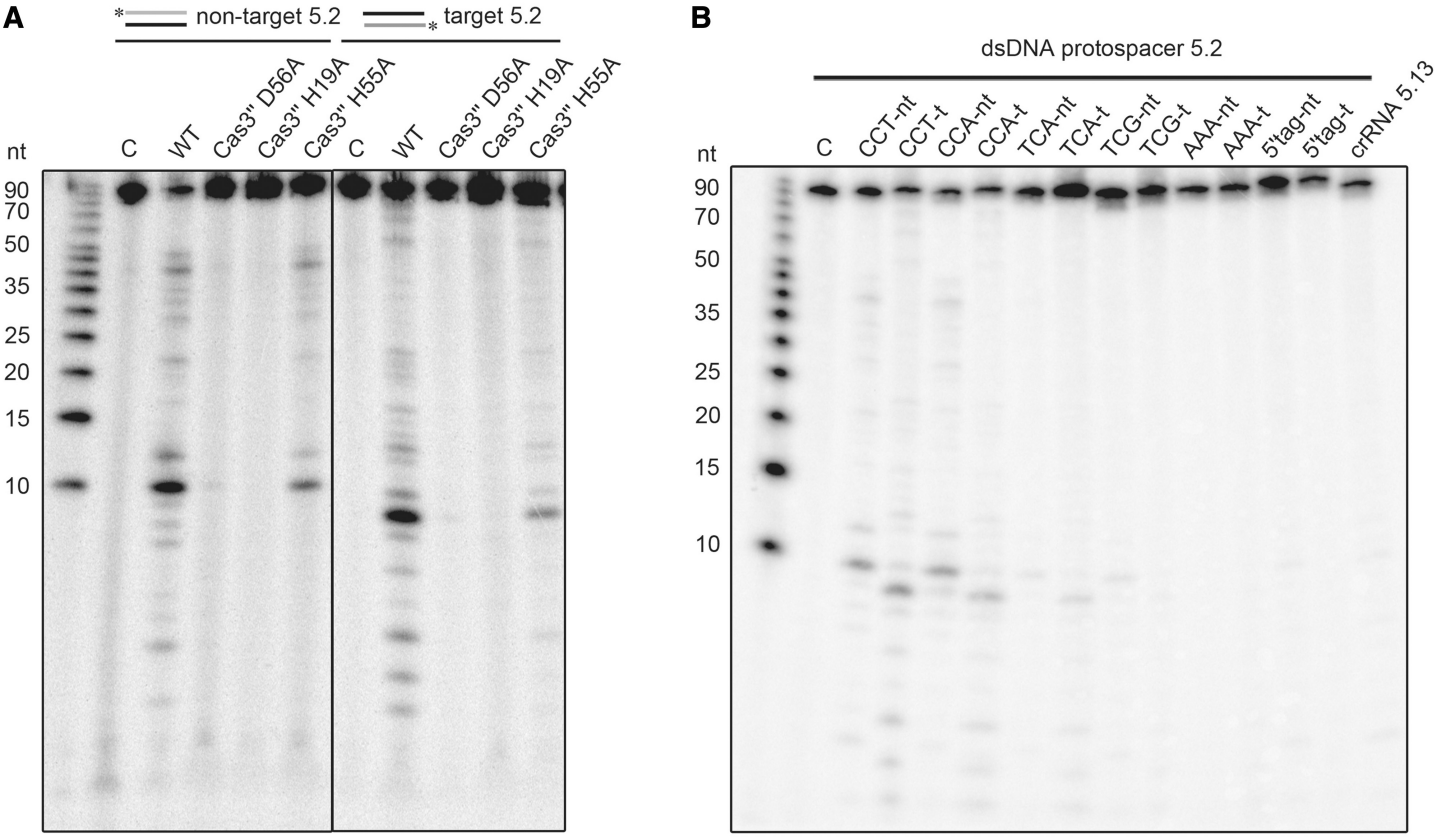
Analysis of Cas3′′ mutants and PAM recognition for *in vitro* assembled type I-A Cascade. (**A**) The Cas3′′-constructed mutants (H19A, H55A, D56A) were assembled into Cascade and tested for dsDNA cleavage. (**B**) The dsDNA substrate 5.2 was mutated to include the indicated 3-bp long PAM sequences (CCT to CCA, TCA, TCG, AAA or a PAM identical to the crRNA 8-nt tag). Cascade-mediated interference reactions were performed with either 5′-[γ-^32^P]-ATP labeled non-target (forward) or the crRNA target strand (reverse) as a substrate, while Cascade was loaded with the spacer matching crRNA 5.2. The loaded non-matching crRNA 5.13 served as a negative control.

The co-refolded Cascade in combination with *in vitro* processed crRNA provides an active interference complex cleaving dsDNA in a RNA sequence-dependent manner. This set up allows the modulation of individual components and to test their influence on Cascade-mediated interference. First, the Cas3′′ mutants H19A, H55A and D56A were assembled into Cascade and tested for their ability to cleave dsDNA substrates (Figure 5A). All three mutants were impaired in dsDNA cleavage. The H19A and D56A mutants showed no activity for the non-target or target strand, while H55A is strongly reduced in dsDNA cleavage. Next, the effect of different PAM sequences on the *in vitro* interference reaction was assayed. It should be noted that Cascade recognizes PAM sequences as so-called target interference motifs that might slightly differ from the PAM sequence recognized during adaptation (56). Owing to missing viral targets of *T. tenax* spacers, the exact PAMs are not known. Detailed analysis of *S. solfataricus* CRISPR systems revealed CCN sequences located 5′ to the DNA strand corresponding to the crRNA (non-target strand) as functional PAMs (10,57), and computational studies identified the TCN motif as a potential PAM (56,58). Therefore, the designed dsDNA protospacer substrate 5.2 was modified upstream in the spacer-adjacent three base pair motif, while the spacer and the flanking sequences remained identical. The tested dsDNA substrates contained the PAM sequences CCT, CCA, TCA, TCG, AAA and a motif identical to the 5′-tag of the crRNA (mimicking self-targeting at the CRISPR locus) (Figure 5B). The interference reaction showed similar Cascade cleavage patterns of dsDNA for the PAM CCT and CCA, confirming that CCN motif is a PAM that allows DNA target cleavage. A reduced activity was observed for the TCA PAM, while substrates with the TCG, AAA or the 5′-tag motif were not cleaved by Cascade.

## DISCUSSION

Type I CRISPR-encoded interference is mediated by a crRNA-guided Cascade complexed with Cas3 to degrade the foreign target DNA. Here, we described the production and assembly of an archaeal type I-A Cascade with co-refolded recombinant Cas proteins and synthetic crRNA transcripts, which resulted in an active complex capable of crRNA-guided degradation of dsDNA in dependence of PAM sequences. 

The analysis of *T. tenax* RNA-Seq read mapping revealed the mature crRNA termini and confirmed previous northern blot analyses, which indicated that *T. tenax* harbors five highly active CRISPR loci and two inactive CRISPR loci (41). These inactive and active clusters contain conserved motifs for transcription initiation and differ mainly in their respective repeat sequences (seven base exchanges and one base length difference). Therefore, it is plausible that this repeat is not processed by the two encoded Cas6 proteins. Accordingly, in type III-A systems, crRNAs or their precursors cannot be detected in Cas6 deletion mutants suggesting degradation of the primary transcript (59). Alternatively, the transcription of the two inactive loci could be regulated by an unknown mechanism. To engineer functional synthetic crRNAs, the *in vivo* processing of mature crRNAs by Cas6 had to be mimicked, as soluble recombinant Cas6 was not available. The technique of fusing a *cis*-acting hammerhead ribozymes to RNA transcripts to obtain defined termini (54,55) was adapted for the production of crRNAs, which resulted in 8 nt tags with 5′-hydroxyl ends (Supplementary Figure S2A and B). This methodology allows the *in vitro* production of crRNAs that start without the guanosine base that is required for proper T7 RNA polymerase transcription initiation. The RNA-Seq data additionally revealed gradually degraded 3′-termini for all crRNAs. The used synthetic crRNAs contained a 2 nt tag (5′-GA-3′), which was observed for the majority of crRNA reads. The trimming of the crRNAs’ 3′-end is typically observed in many different bacteria and archaea, but the exact mechanism is not known (60,61). It is likely that Cas6-processed crRNAs are protected by Cascade proteins binding to the conserved 5′-end, whereas the free 3′-ends are unspecifically trimmed by cellular RNases due to a missing secondary structure or that crRNAs are unstable at elevated temperatures (62,63). For organisms with multiple encoded CRISPR-Cas effector complexes, distinct crRNA species with varying 3′-ends might be sorted between complexes in dependence of their length (64). These potential parameters for crRNA loading into recombinant Cascade could be tested by engineering multiple modified synthetic RNAs. Other possible applications for the use of synthetic crRNA transcripts are dye-labeled RNA constructs for smFRET measurements or crRNA-Cascade cross-linking with photoreactive nucleotide analogs that facilitates detailed information about the RNA structure and motion during complex formation and a precise mapping of the cross-link between crRNA and Cas protein subunit (65–67). 

Previous research focused on the detailed analysis of the *E. coli* type I-E Cascade function and structure (19,31). One main feature of the type I-E interference mechanism is a separation into the Cascade module that is binding the crRNAs recognizing the matching DNA target and the Cas3 protein, which is recruited by Cascade to the crRNA:dsDNA R-loop structure. The *E. coli* Cascade is built up by the subtype specific subunits Cse1 and Cse2 that are proposed to interact with the DNA target, the conserved crRNA binding subunits Cas7 and Cas5e and the pre-crRNA cleaving endoribonuclease Cas6e (18,29,30). From a mechanistic point of view, this arrangement guarantees a streamlined processing of the pre-crRNA transcripts, immediate crRNA protection and the scanning for the complementary DNA target, localized within one protein complex. Additionally, the transcription of pre-crRNAs and *cascade* genes are strictly repressed by the global regulator H-NS and activated with the help of the transcription factor LeuO, ensuring a fast response to a viral attack (68–70).

In this study, we analyzed an archaeal type I-A CRISPR-Cas system, which showed remarkable differences in the assembly of Cascade and the crRNA production. A typical feature found in archaeal genomes is the splitting of the two Cas3 domains into separate genes (Cas3′: helicase domain, Cas3′′: nuclease domain) and a gene organization into one *cascade* genes operon that can be regulated by changes of the environmental parameters (41). Similar to type I-E Cascade, the crRNA binding subunits *cas7* and *cas5a* and two subtype-specific subunits *csa5* and *cas8a2* are encoded within the operon. However, two *cas6* genes are encoded separately at distant locations in the genome. Pull-down assays supported that the genomic *cas* gene organization mirrors the Cas protein assembly, as both Cas3 subunits, Cas7, Cas5a and Cas8a2 were interacting with Csa5. The Cas6 protein could not be identified (Supplementary Table SII). In agreement, the co-refolding of all possible protein combinations showed the necessity of Cas3 for the formation of a stable Cascade complex, indicating that the two Cas3 subunits are not recruited but are rather an integral part of the Cascade I-A complex. In contrast to type I-E systems, pre-crRNA transcription and crRNA production appears to be constitutive, as evidenced by the high abundance of crRNAs detectable in RNA-Seq data. Therefore, regulation of Cascade immunity appears to rely on the activation of Cas protein production. The transcription of the Cascade genes might be activated by the encoded regulator protein Csx1 (Csa3), which is followed by assembly of Cascade:Cas3 and immediately loaded with crRNA to cleave the protospacer DNA target (71). A possible reason for the differences in Cascade assembly might be the thermophilic lifestyle of *T. tenax*, which would make a later recruitment of Cas3 to Cascade assembled in an R-loop structure under elevated temperatures challenging. Interestingly, type I-A systems are exclusively found in thermophilic organisms, supporting the evolutionary conservation of this alternative Cascade formation strategy (6).

The established assembly of the *T. tenax* Cascade allowed us to investigate the type I-A interference reaction and the role of individual subunits in more detail. In Cascade nuclease assays, Cas3′′ was identified as the primary deoxyribonuclease subunit, cleaving linear ssDNA substrates exonucleolytically in the dependence of the divalent Mg^2+^ and Mn^2+^ metal ions. Mutations in the conserved HD domain motif (H19A, H55A and D56A) inhibited the cleavage activity. The large group of HD domain proteins comprises enzymes that are primarily involved in nucleic acid metabolism and signal transduction and react on a broad range of substrates, including ssDNA, RNA and R-loop structures (37,72). The degradation of ssDNA substrates by the Cas3′′ subunit was previously characterized for different Cascade subtypes from *M. jannaschii*, *S. thermophilus* or *Thermus thermophilus*, and a crystal structure revealed the active site with two bound metal cations (37–39). The observed unspecific cleavage of ssDNA by Cas3′′ *in vitro* would pose a problem within the cell. The addition of non-matching synthetic crRNA to the nuclease assays indicated an inhibition of this background cleavage activity. This suggests that Cascade might first bind crRNA, which inhibits the unspecific ssDNA cleavage until the correct dsDNA target is specifically located. Another example for the interplay between Cascade subunits was observed for the binding of crRNAs. The purification procedure, EMSA assays and TEM imaging confirmed that *T. tenax* Cas7 binds unspecifically to both crRNAs and other small RNA contaminants. This interaction resulted in the formation of long helical Cas7 multimer structures. The observation of helical Cas7 filaments was also made for a Cascade sub-complex of the subunits Cas7 and Cas5a of *S. solfataricus* (43). The cryo-electron microscopy structure of the I-E Cascade revealed a seahorse-like shape, in which six copies of the Cas7 subunit are forming the backbone, combined to a Cascade stoichiometry of 1× Cse1, Cas5e, Cas6e, 2× Cse2 and 6× Cas7 (18,31). The binding behavior of *T. tenax* Cas7 embedded in I-A Cascade changed significantly. The shifting of crRNA appeared lower during EMSA assays, the gel filtration showed no formation of extended Cas7 multimers and no helical structures were observed in TEM pictures. These observations indicate a coordinated assembly of I-A Cascade and a capping mechanism by other Cascade subunits that block Cas7 from forming extended multimeric filaments. The exact stoichiometry for I-A Cascade could not be determined as the amount of functional refolding is not known. Future work on the Cascade structure via TEM, crystallization or native mass spectrometry is required to address these questions. 

In interference assays the assembled I-A Cascade exhibited crRNA-mediated cleavage of dsDNA molecules. In the targeting event, Cascade facilitates base pairing of the crRNA with the complementary target strand and additional displacement of the non-target strand to produce the R-loop structure (33,35). An open question is the nature of the crRNA seed sequence specifically for the type I-A CRISPR-Cas system, defined as the minimal sequence complementarity of crRNA and target DNA for binding (32,73). The existence of an adjacent PAM was shown to be essential for directing DNA cleavage (57). Functional PAM sequences for the *T. tenax* I-A Cascade were CCA or CCT, whereas other flanking sequences yielded a reduced (TCA) or impaired activity (TCG). The origin of the *T. tenax* spacers is not known. Therefore, the established interference assay allowed us to identify this basic requirement for Cascade-mediated cleavage and can be used to complement plasmid-based *in vivo* assays that determined the PAM sequences *in vivo* for I-A and I-B systems (11,56,74,75). The main function of the PAM is the discrimination between host CRISPR loci DNA and protospacer/target sequences. Cascade first screens for the specific PAM followed by a helical destabilization and strand invasion of the crRNA, which leads to the R-loop formation (61,76). An outstanding question is the mechanism for the identification of the correct PAM, which triggers the crRNA-loaded Cascade for targeting. Two candidates Csa5 and Cas8a2 that are described as the small and large subunit in Cascade might interact with the DNA target and/or recognize the PAM (5,77). The Cascade-mediated interference assays reflect the stepwise degradation of the dsDNA, as Cas3′′ first cleaves in the middle of the looped out non-target strand (at position 43–47 nt) followed by a gradual cleavage in 3′–5′ direction. In a second step, the target strand is then cleaved over the entire length also in 3′–5′ direction, which was reported previously for other members of the Cas3 family (37–39). The identified minimal cleavage products are similar to ones found in the type I-E system with 8–10-nt fragment length, which suggests a conserved cleavage mechanism (35).

In conclusion, the established *in vitro* assembly and interference activity protocols of a type I-A Cascade with synthetic crRNA molecules highlight similarities and differences between this archaeal interference complex and the type I-E Cascade. These studies are expected to aid in the assembly of other Cascade complexes to help us to understand commonalties and differences in the evolution of the diverse type I Cascade machineries.

## SUPPLEMENTARY DATA

Supplementary Data are available at NAR Online.

## FUNDING

Deutsche Forschungsgemeinschaft [DFG, FOR1680] and the Max-Planck Society. Funding for open access charge: [DFG, FOR1680, Max-Planck Society].

*Conflict of interest statement*. None declared.

## Supplementary Material

Supplementary Data
