# Supplementary material for: In vitro assembly and activity of an archaeal CRISPR-Cas type I-A Cascade interference complex
Source: Nucleic Acids Res. 2014 Feb 5;42(8):5125–38. doi: 10.1093/nar/gku120 (PMC4005679; doi:10.1093/nar/gku120)
Supplement: Supplementary Data [file supp_42_8_5125__index.html]

In vitro assembly and activity of an archaeal CRISPR-Cas type I-A Cascade interference complex — In vitro assembly and activity of an archaeal CRISPR-Cas type I-A Cascade interference complex — Supplementary Data 

# *In vitro* assembly and activity of an archaeal CRISPR-Cas type I-A Cascade interference complex

## Supplementary Data

files

**Files in this Data Supplement:**

- Supplementary Data - pdf file
