# Supplementary material for: In vitro assembly and activity of an archaeal CRISPR-Cas type I-A Cascade interference complex
Source: Nucleic Acids Res. 2014 Feb 5;42(8):5125–38. doi: 10.1093/nar/gku120 (PMC4005679; doi:10.1093/nar/gku120)
Supplement: Supplementary Data [file supp_gku120_nar-03645-h-2013-File007.pdf]

SUPPLEMENTARY DATA

SUPPLEMENTARY FIGURES

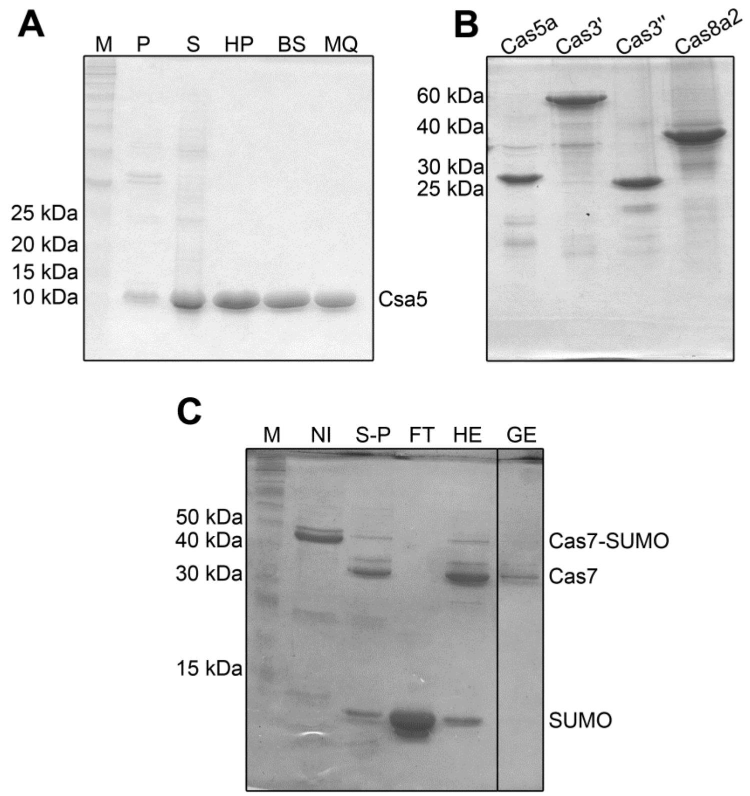

Fig. S1: Protein purification of all six Cascade subunits. (**A**) The soluble protein Csa5 was purified via a two-column strategy. Depicted are the pellet (P) and supernatant (S) fractions after cell lysis and ultracentrifugation, followed by the heat precipitated (HP, 90°C) Csa5 sample, further purified samples via Blue Sepharose affinity chromatography (BS) and MonoQ anion-exchange chromatography (MQ) on a 15% SDS gel next to the protein marker (M). (**B**) The insoluble subunits Cas5a, Cas3', Cas3'' and Cas8a2 were individually purified via inclusion body isolation and solubilization in 6 M GdmCl. The 15% SDS gel shows the subunits before usage in Cascade assembly. (**C**) The soluble subunit Cas7 was fused with a SUMO tag to omit cross-contamination with *E. coli* RNA and oligomerization. Depicted are the individual protein fractions alongside the protein marker (M) separated on a 15% SDS-PAGE. Cas7-SUMO was purified via Ni-NTA chromatography (NI), followed by a SUMO protease (S-P) treatment during dialysis. After concentration of native Cas7 (centrifugal filter units, MWCO: 30 kDa) the cleaved off SUMO tag was visible in the flow-through (FT). Cas7 was further purified with Heparin cation-exchange chromatography (HE) and gel filtration (GE) resulting in monomeric Cas7.

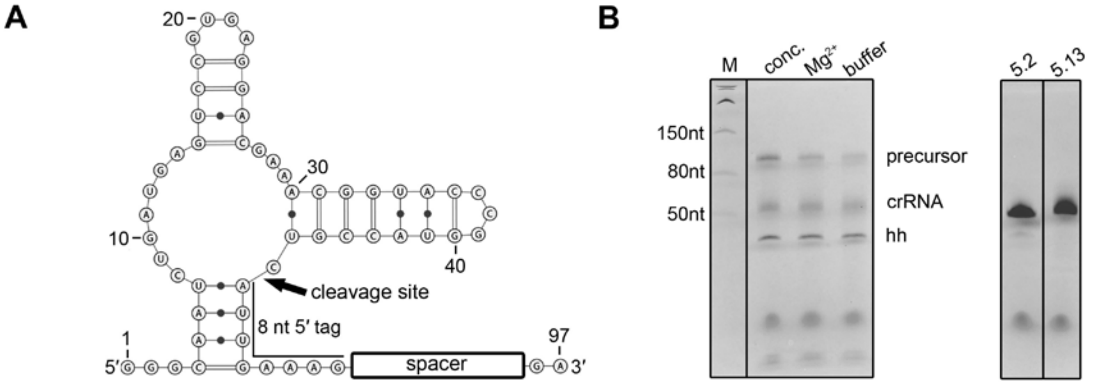

Fig. S2: Production of synthetic crRNAs. (**A**) Mature crRNAs 5.2 and 5.13 were produced via fusion of the crRNA sequence to the sequence of a minimal *cis*-acting hammerhead (hh) ribozyme, resulting in a self-cleavage (arrow marking the cleavage site) directly upstream of the 8 nt 5'-tag. (**B**) The self-cleavage of the transcript (precursor) into crRNA and hh was induced by a direct incubation at 60°C (conc.), dilution of the sample in 4 vol of 30 mM $MgCl_2$ in DEPC-H_2O (Mg^{2+}) or dilution in 4 vol of *in vitro* transcription buffer (buffer) followed by heat incubation, yielding the synthetic mature crRNAs 5.2 (50 nt) and 5.13 (54 nt).

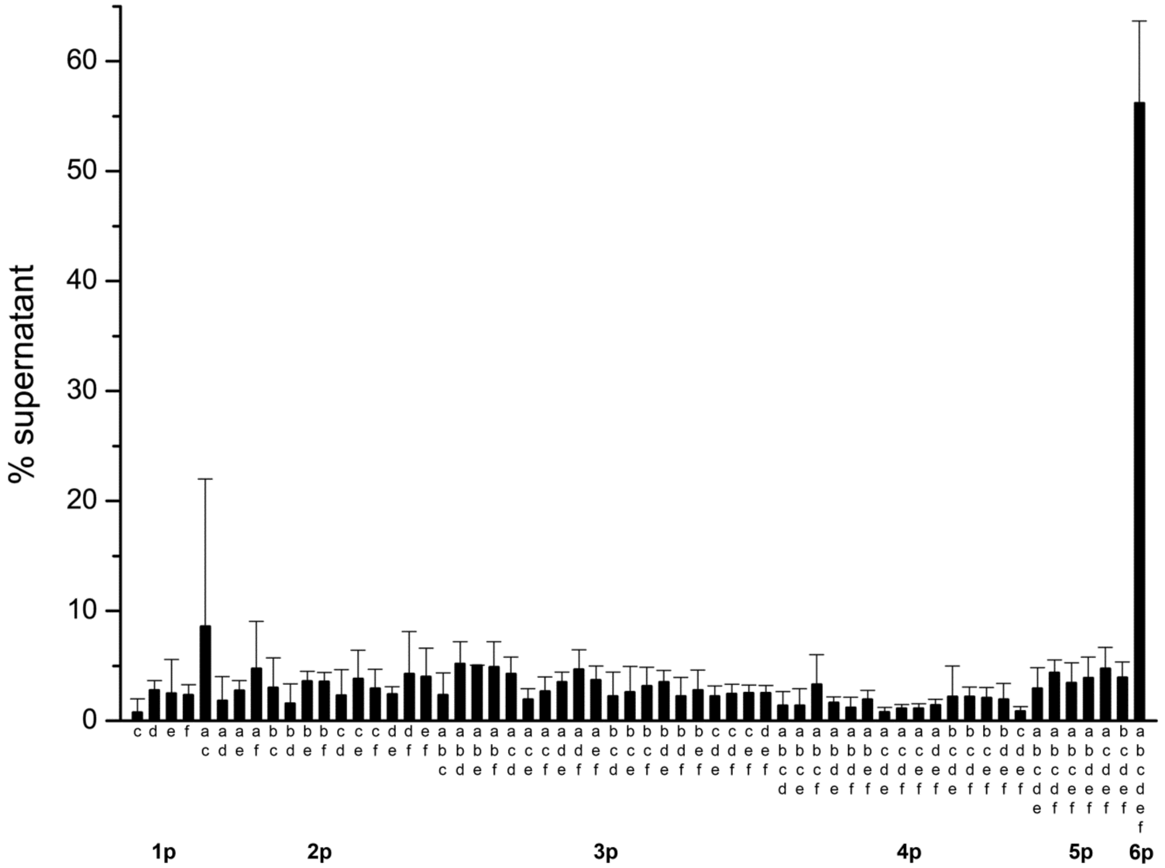

Fig. S3: Co-refolding combinations of Cascade subunits. All possible 63 combinations of Cascade subunits were reconstituted by the rapid dilution method and for each protein, the obtained bands on the SDS gel compared between soluble protein (supernatant) and aggregated protein (pellet). Plotted is the average of co-refolded supernatant (%) of 60 combinations that contained insoluble proteins (c: Cas5a, d: Cas3', e: Cas3'', f: Cas8a2), while the soluble proteins (a: Csa5, b: Cas7) are not taken into account. Only the combination that contained all six Cascade subunits (6p) showed a recovery of soluble protein above 50%, in contrast to combinations with missing subunits (1p-5p) that resulted in soluble protein of less than 10%.

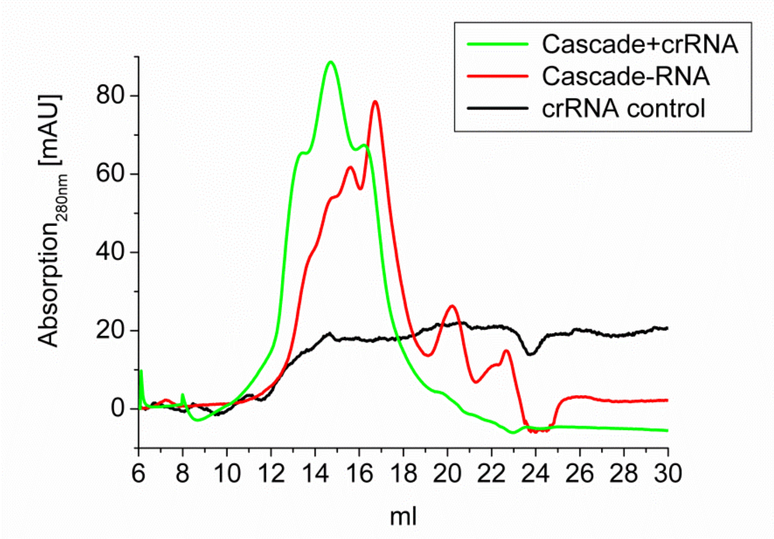

Fig. S4: Size-exclusion chromatograms of assembled Cascade. The Cascade subunits (Csa5, Cas7, Cas5a, Cas3', Cas3'' and Cas8a2) were assembled via the co-refolding procedure and incubated with synthetic crRNA 5.2 (crRNA) or no RNA (-RNA) and protein was followed via absorption at 280 nm. A protein-free sample of the crRNA served as a control of the elution profile (crRNA control). The chromatograms demonstrate that protein is eluting in the fractions 10-18 ml (Fig. 2A).

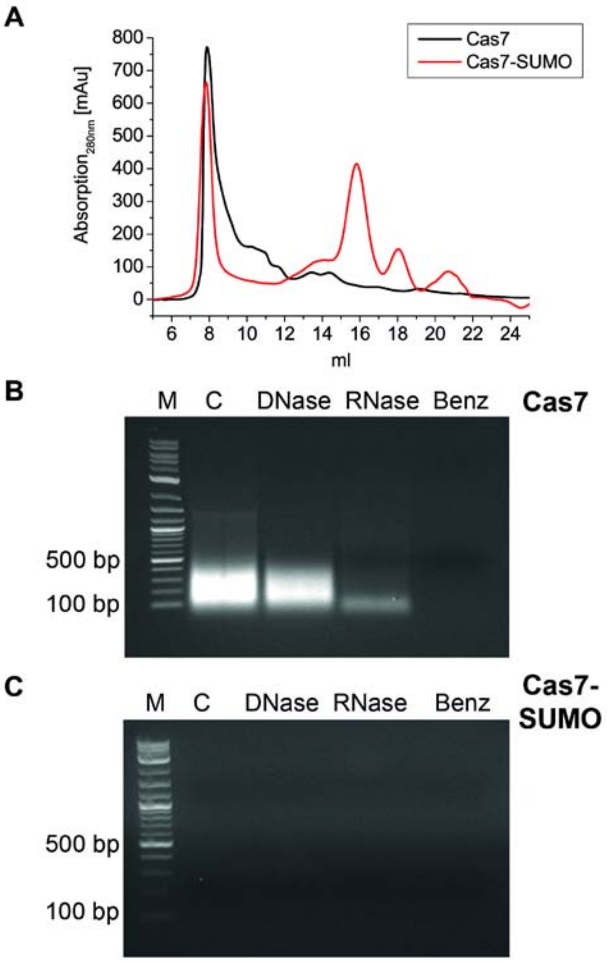

Fig. S5: Comparison of Cas7 and Cas7-SUMO purification procedures. (**A**) The two Cas7 versions (native purification or SUMO-tagged) exhibit different elution profiles on size-exclusion columns. The native purification of Cas7 results in completely assembled large multimeric complexes (black line, elution volume: 8.2 ml), while the Cas7-SUMO version elutes after cleavage of the SUMO-tag as a monomer (red line, elution volume: 15.5 ml). (**B**) 100 µg of Cas7 loaded on a 1% agarose gel and stained with EtBr next the DNA marker shows cross-contamination of *E. coli* RNA (C), which are cleaved by RNase A or Benzonase, but not by DNaseI. (**C**) The purification of Cas7-SUMO is free of any cross-contamination, which is shown by loading identical amounts of protein on a 1% agarose gel.

TEM: Cas7+RNA

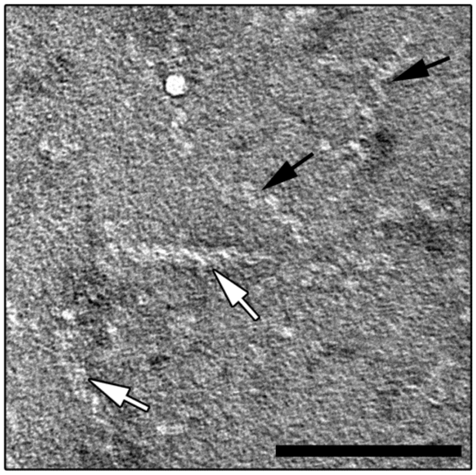

Fig. S6: Helical artifact formation of Cas7 with *E. coli* RNA. A typical TEM analysis of a Cas7 purification resulted in cross-contamination with *E. coli* RNA and the formation of helical (black arrows) and double-helical filaments (white arrows) of up to 50 nm length. Scale bar: 50 nm.

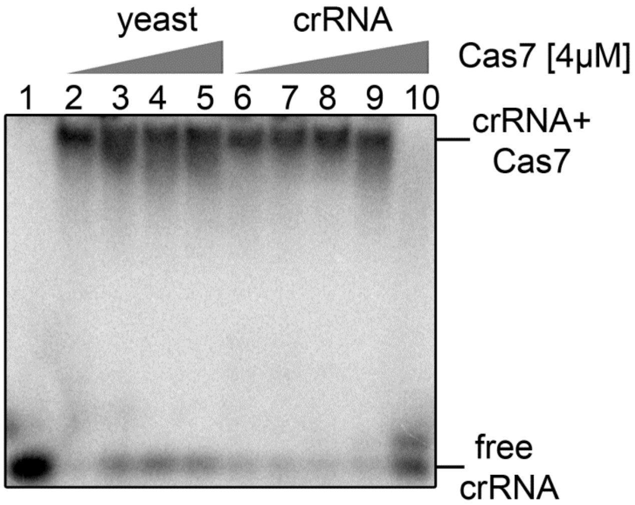

Fig. S7: Specificity of Cas7 binding to crRNA. At a fixed Cas7 concentration of 4 µM the specificity of crRNA binding (crRNA 5.2) was tested by competing amounts of yeast RNA (lane 2-6: 0, 10, 50, 250, 500 ng) or increasing amounts of unlabeled crRNA 5.2 (lane 7-10: 1:1, 1:10, 1:100, 1:1000). Only the highest concentration of unlabeled crRNA abolished the Cas7 shift indicating an unspecific binding of Cas7 to crRNA. Lane 1 serves as a crRNA loading control.

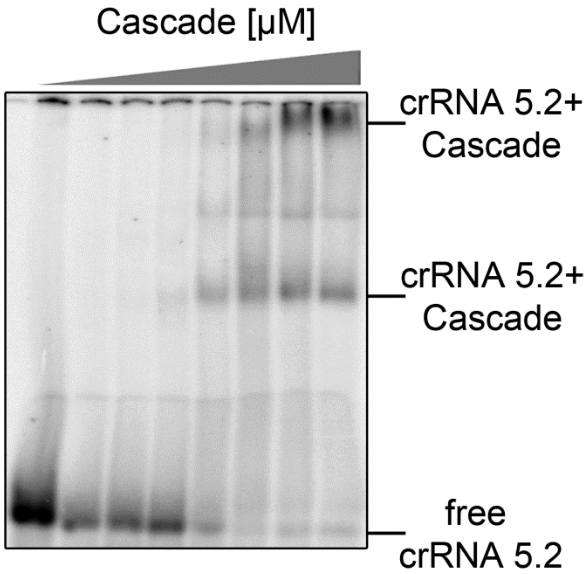

Fig. S8: Cascade binding to the synthetic crRNA 5.2. The assembled Cascade was tested in EMSAs for specific binding to the constructed crRNA 5.2. Increasing concentrations (0, 0.125, 0.25, 0.5, 1, 2, 4, 5 µM) of Cascade in the presence of yeast RNA showed a shifting of the 5'-labeled (with [γ-^{32}P]-ATP) crRNA 5.2 on 6% non-denaturing gels comparable to the synthetic crRNA 5.13.

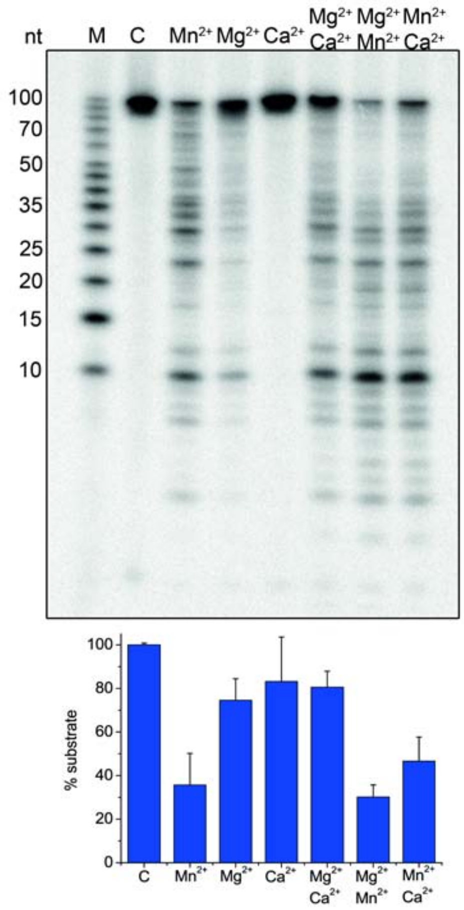

Fig. S9: The cleavage of ssDNA by Cascade is metal-dependent. Cascade was incubated with the 5'-[γ-^{32}P]-ATP labeled short ssDNA substrate (int 5.2_CCT for) and different metal combinations for 10 min at 70°C and the cleavage products were separated on a 15% denaturing gel next to the low molecular weight marker. The assay was repeated three times and in each reaction the remaining substrate estimated via line profile plots (Image J), which indicated that Cascade in the combination with Mg^{2+} and Mn^{2+} ions has the highest activity (~70% cleaved) on ssDNA.

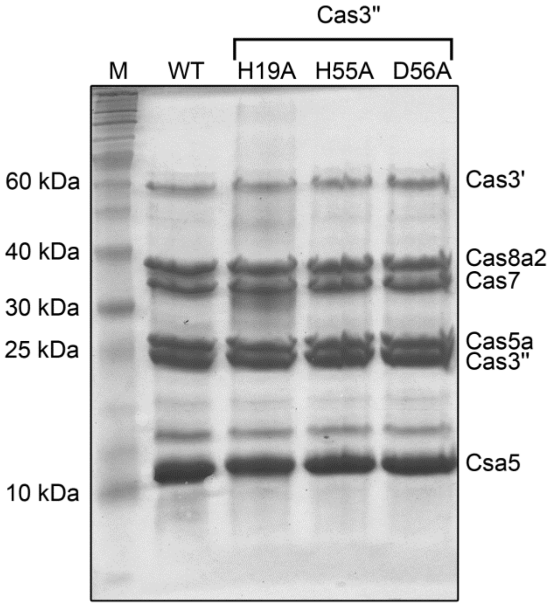

Fig. S10: Co-reconstitution of Cascade with wild-type Cas3'' and Cas3'' mutants. All Cascade complexes were assembled via the same co-refolding procedure of insoluble proteins including either Cas3'' WT, Cas3'' H19A, Cas3'' H55A or Cas3'' D56A. A 15 µl sample of the supernatant after Cascade co-refolding into native buffer, centrifugation and concentration was loaded on the 15% SDS gel alongside the protein marker (M), demonstrating the same reconstitution efficiency for all Cas3'' proteins within Cascade.

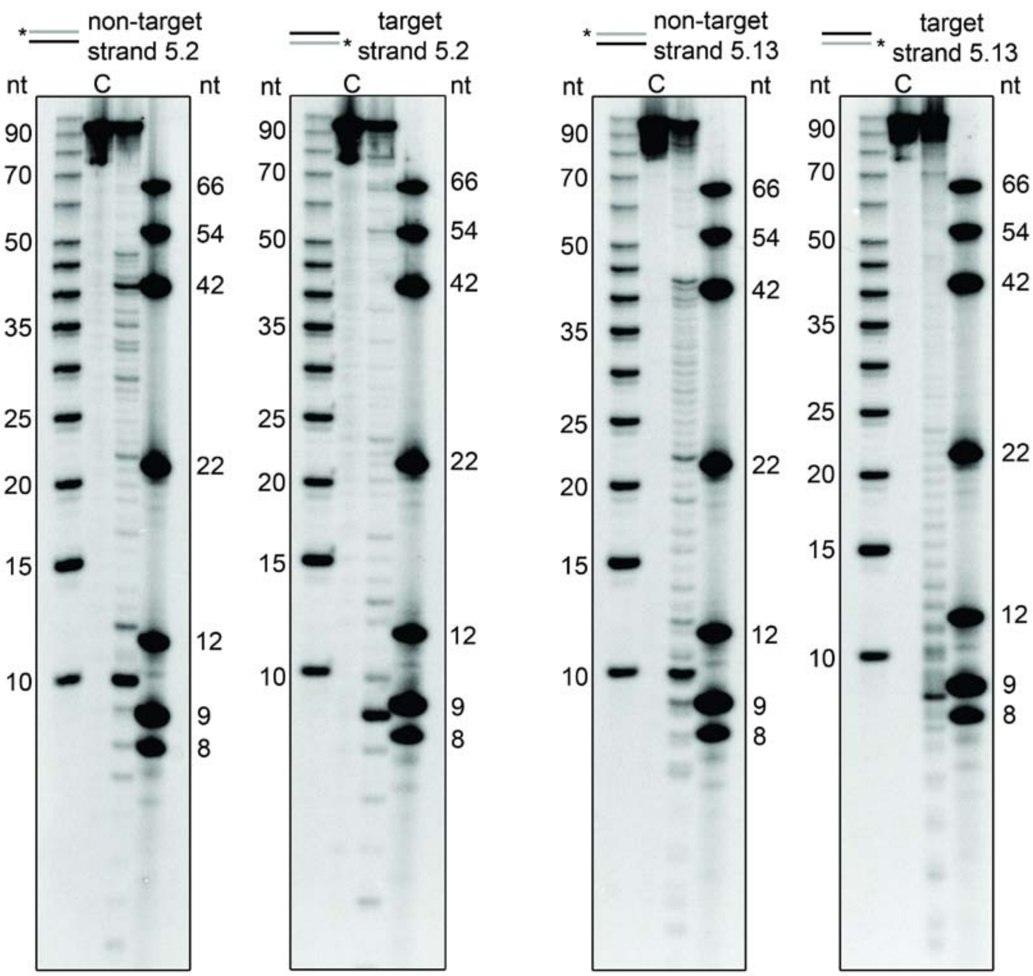

Fig. S11: Size determination of the cleavage products appearing in the Cascade interference reaction. The 5'-[γ-^{32}P]-ATP labeled non-target and target strand of each dsDNA substrate 5.2 or 5.13 were separated on 10% denaturing sequencing gels alongside the 5'-labeled low molecular weight marker (10-100 nt) or a mixture of seven ssDNA fragments (8-66 nt) to specify the fragment length of cleavage hot spots. Lanes marked with C are the loading controls of the dsDNA.

SUPPLEMENTARY TABLES

Table SI: Oligonucleotides and RNA sequences for cloning and substrate generation

name	sequence
cr5.2h (125bp)	BamHI – T7 promoter – hammerhead – crDNA, 5.2 – HindIII
cr5.2h for (119nt)	GATCCTAATACGACTCACTATAGGGCAATCTGATGAGTCCGTGAGGACGAAACGGTACCCGGTACCGTCATTGAAAGCGTTGATGCGGCCGCGACTGGCTGACTCAGCTATTACGTTGA
cr5.2h rev(119nt)	AGCTTCAACGTAATAGCTGAGTCAGCCAGTCGCGGCCGCATCAACGCTTTCAATGACGGTACCGGGTACCGTTTCGTCCTCACGGACTCATCAGATTGCCCTATAGTGAGTCGTATTAG
cr5.13h (126bp)	HindIII – T7 promoter – hammerhead – crDNA, 5.13 – EcoRI
cr5.13h for (120nt)	AGCTTAATACGACTCACTATAGGCAATCTGATGAGTCCGTGAGGACGAAACGGTACCCGGTACCGTCATTGAAAGATCGCTTGTCCAACCGGGCTCCTCTATATGTCGTCATTAGCTTAG
cr5.13h rev (120nt)	AATTCTAAGCTAATGACGACATATAGAGGAGCCCGGTTGGACAAGCGATCTTTCAATGACGGTACCGGGTACCGTTTCGTCCTCACGGACTCATCAGATTGCCTATAGTGAGTCGTATTA
cr5.2PCRf (19nt)	GGGGATCCTAATACGACTC
cr5.2PCRr (20nt)	TCAACGTAATAGCTGAGTCA
cr5.13PCRf (19nt)	GCCAAGCTTAATACGACTC
cr5.13PCRr (20nt)	TCTAAGCTAATGACGACATA
crRNA 5.2 (50nt)	AUUGAAAGCGUUGAUGCGGCCGCGACUGGCUGACUCAGCUAUUACGUUGA
crRNA 5.13 (54nt)	AUUGAAAGAUCGCUUGUCCAACCGGGCUCCUCUAUAUGUCGUCAUUAGCUUAGA
int 5.2 (93bp)	SalI – 5'-proto – PAM (NNN) – spacer 5.2 – 3'-proto – HindIII
int 5.2_CCT for (93nt)	GTCGACTAATACGACTCACTATAGACCTCGTTGATGCGGCCGCGACTGGCTGACTCAGCTATTACGTTCCGCTGAGCAATAACTAGCAAGCTT
int 5.2_CCT rev (93nt)	AAGCTTGCTAGTTATTGCTCAGCGGAACGTAATAGCTGAGTCAGCCAGTCGCGGCCGCATCAACGAGGTCTATAGTGAGTCGTATTAGTCGAC
int 5.2_CCA for (93nt)	GTCGACTAATACGACTCACTCGCAGCCACGTTGATGCGGCCGCGACTGGCTGACTCAGCTATTACGTTCCGCTGAGCAATAACTAGCAAGCTT
int 5.2_CCA rev (93nt)	AAGCTTGCTAGTTATTGCTCAGCGGAACGTAATAGCTGAGTCAGCCAGTCGCGGCCGCATCAACGTGGCTGCGAGTGAGTCGTATTAGTCGAC
int 5.2_TCA for (93nt)	GTCGACTAATACGACTCACTCGCAGTCACGTTGATGCGGCCGCGACTGGCTGACTCAGCTATTACGTTCCGCTGAGCAATAACTAGCAAGCTT
int 5.2_TCA rev (93nt)	AAGCTTGCTAGTTATTGCTCAGCGGAACGTAATAGCTGAGTCAGCCAGTCGCGGCCGCATCAACGTGACTGCGAGTGAGTCGTATTAGTCGAC
int 5.2_TCG for (93nt)	GTCGACTAATACGACTCACTCGCAGTCGCGTTGATGCGGCCGCGACTGGCTGACTCAGC

	TATTACGTTCCGCTGAGCAATAACTAGCAAGCTT
int 5.2_TCG rev (93nt)	AAGCTTGCTAGTTATTGCTCAGCGGAACGTAATAGCTGAGTCAGCCAGTCGCGGCCGCA TCAACGCGACTGCGAGTGAGTCGTATTAGTCGAC
int 5.2_AAA for (93nt)	GTCGACTAATACGACTCACTCGCAGAAACGTTGATGCGGCCGCGACTGGCTGACTCAGC TATTACGTTCCGCTGAGCAATAACTAGCAAGCTT
int 5.2_AAA rev (93nt)	AAGCTTGCTAGTTATTGCTCAGCGGAACGTAATAGCTGAGTCAGCCAGTCGCGGCCGCA TCAACGTTTCTGCGAGTGAGTCGTATTAGTCGAC
int 5.2_Rep for (98nt)	GTCGACTAATACGACTCACTATAGAATTGAAAGCGTTGATGCGGCCGCGACTGGCTGAC TCAGCTATTACGTTCCGCTGAGCAATAACTAGCAAGCTT
int 5.2_Rep rev (98nt)	AAGCTTGCTAGTTATTGCTCAGCGGAACGTAATAGCTGAGTCAGCCAGTCGCGGCCGCA TCAACGCTTTCAATTCTATAGTGAGTCGTATTAGTCGAC
int 5.13 (97bp)	SalI – 5'-proto – PAM (CCT) – spacer 5.13 – 3'-proto – HindIII
int 5.13_CCT for (97nt)	GTCGACTAATACGACTCACTATAGGCCTATCGCTTGTCCAACCGGGCTCCTCTATATGT CGTCATTAGCTTACCGCTGAGCAATAACTAGCAAGCTT
int 5.13_CCT rev (97nt)	AAGCTTGCTAGTTATTGCTCAGCGGTAAGCTAATGACGACATATAGAGGAGCCCGGTTG GACAAGCGATAGGCCTATAGTGAGTCGTATTAGTCGAC
Csa5-His for (20nt)	TCCTAATACGACTCACTATA
Csa5-His rev (23nt)	GGAGCCACCCAAGCTTCCCCTTA
Cas3''-H19A for (34nt)	CCAGACCTACGAAGACGCCATCACGCAGGCTCTG
Cas3''-H19A rev (34nt)	CAGAGCCTGCGTGATGGCGTCTTCGTAGGTCTGG
Cas3''-H55A for (32nt)	CTAGCCGTGGAGTTCGCCGACCTAGGCAAGCT
Cas3''-H55A rev (32nt)	AGCTTGCCTAGGTCGGCGAACTCCACGGCTAG
Cas3''-D56A for (29nt)	CGTGGAGTTCCACGCCCTAGGCAAGCTCG
Cas3''-D56A rev (29nt)	CGAGCTTGCCTAGGGCGTGGAACTCCACG
MI_8 (8nt)	CATCAACG
MI_9 (9nt)	GCATCAACG
MI_12 (12nt)	GCCGCATCAACG
MI_22 (22nt)	GCCAGTCGCGGCCGCATCAACG
MI_42 (42nt)	GCATCTAATACGACTCACTATAGGGAGCGAATGAAACGAGCG
MI_54 (54nt)	GCAGCACTCGAGCAATTGTTACACGAAACCTTTACCCACACGTTCCACGGTGCC
MI_66 (66nt)	AGCTTTAATACGACTCACTATAGATTAATCCCATAATACTTTTCTAGGTCTGGGCGGAA TGGATCC

Table SII: MS analysis of *in vivo* pull-down Cascade experiments. Proteins co-purifying with Csa5 were identified by *in solution* trypsin digestion and followed by MS.

**ORF**	**protein**	**1) intensity**	**1) coverage [%]**	**2) intensity**	**2) coverage [%]**
TTX_1250	Csa5	8.46E+10	96.2	6.22E+10	96.2
TTX_1251	Cas7	-	-	836940	7.9
TTX_1252	Cas5a	-	-	-	3.5
TTX_1253	Cas3'	30243000	14	134950	1.2
TTX_1254	Cas3''	-	-	71119	4.9
TTX_1255	Cas8a2	12116000	14.4	-	-
TTX_0235	Cas7	27699000	17	138670	3.2
